# Caspase-1-Dependent Pyroptosis Mediates Adjuvant Activity of Platycodin D as an Adjuvant for Intramuscular Vaccines

**DOI:** 10.3390/cells11010134

**Published:** 2022-01-01

**Authors:** Liyan Zhu, Ziyi Han, Yanfei He, Hongxiang Sun

**Affiliations:** Department of Veterinary Medicine, College of Animal Sciences, Zhejiang University, Hangzhou 310058, China; juliet@zju.edu.cn (L.Z.); 15222671162@163.com (Z.H.); 11717039@zju.edu.cn (Y.H.)

**Keywords:** platycodin D, adjuvant, pyroptosis, caspase-1, JNK and p38 MAPK signaling, inflammatory response

## Abstract

Platycodin D (PD) is a potent adjuvant with dual Th1 and Th2 potentiating activity, but its mechanisms of action remain unclear. Here, the C2C12 myoblast cell line and mice were used as in vitro and in vivo models to identify potential signaling pathways involved in the adjuvant activity of PD. PD induced a transient cytotoxicity and inflammatory response in the C2C12 cells and in mouse quadricep muscles. A comparative analysis of microarray data revealed that PD induced similar gene expression profiles in the C2C12 cells and in the quadricep muscles, and triggered rapid regulation of death, immune, and inflammation-related genes, both in vivo and in vitro. It was further demonstrated that caspase-1-dependent pyroptosis was involved in the PD-induced cytotoxicity and inflammatory response in the C2C12 cells via the Ca^2+^–c-jun N-terminal kinase (JNK)/p38 mitogen-activated protein kinase (MAPK)–NLR family pyrin domain containing 3 (NLRP3) inflammasome signaling pathway. Consistently, the in vivo analysis revealed that a local blockage of NLRP3 and caspase-1 inhibited PD-induced cytokine production and immune cell recruitment at the injection site, and impaired the adjuvant activity of PD on antigen-specific immune responses to model antigen ovalbumin (OVA) in mice. These findings identified the caspase-1-dependent adjuvanticity of PD and expanded the current knowledge on the mechanisms of action of saponin-based adjuvants.

## 1. Introduction

An adjuvant, an essential component of the new-generation vaccine, stimulates innate immunity and shapes the adaptive immune response that eventually confers protection against pathogens [1]. However, very few adjuvants have been licensed for clinical use, owing to their lower potency, severe side effects, limited understanding of their mechanisms of action, and a lack of druggable targets [2,3,4].

QS-21 from *Quillaja saponaria* tree bark is the most known saponin adjuvant. In view of its unique capacity to stimulate both humoral and cellular immune responses, QS-21 has been extensively investigated in clinical trials of therapeutic cancer vaccines, as well as the vaccines directed against intracellular pathogens [5,6]. However, QS-21 has serious drawbacks, such as swelling and erythema at the injection site, adjuvant-inactive and hemolytic byproducts by spontaneous hydrolysis of the acyl chain ester linkages in the aqueous phase [7], as well as low yielding and heterogeneity [8]. Indeed, QS-21 is not a single molecule but a ≈ 2:1 mixture of two isomeric constituents, QS-21-Api and QS-21-Xyl [9].

Platycodin D (PD) is a saponin monomer compound from the roots of *Platycodon grandiflorum* A. DC [10,11]. PD has been proven to improve both antigen-specific cellular and humoral immune responses, and simultaneously elicits a Th1/Th2 response to the recombinant hepatitis B antigen and the Newcastle disease virus-based recombinant avian influenza vaccine in mice [12,13]. PD could be a promising adjuvant candidate with lower hemolysis and toxicity, as well as excellent stability in the aqueous phase.

The studies on the mechanism of saponin-based adjuvants (SBAs) were focused on their effects on the immune cells, especially antigen-presenting cells (APCs). It was reported that a sufficient activation of APCs was particularly crucial for innate and adaptive immunity by SBAs [14,15,16,17,18]. However, the inflammasome activation in APCs by QS-21 decreased human immunodeficiency virus (HIV) gp120/QS-21 vaccine efficacy in vivo [18]. These controversial results can be partly attributed to the lack of suitable in vitro models.

Most vaccines are usually administered by intramuscular injection in a clinic. The immune cells are relatively few and the muscle cells dominate in muscle tissues. Most of the cells exposed to the adjuvant are muscle fibers. It was reported that MF59 induced the activation of muscle fibers at the injection site and that muscle fibers might be the main target of MF59 [19,20]. The expression of Toll-like receptors (TLRs), cytokine receptors, adhesion molecules, costimulatory molecules, and the major histocompatibility complex have been demonstrated in muscle cells in vivo and in vitro [21,22,23], providing a molecular basis for their response to environmental elements, including pathogens and stimuli [24,25]. The skeletal muscles were also reported to secrete a host of cytokines and chemokines, such as interleukin (IL)-6, IL-1α, IL-1β, C-C motif chemokine ligand (CCL) 3, CCL4, and C-X-C motif chemokine ligand (CXCL) 2, which played a pivotal role in maintaining and amplifying the local inflammatory response [26,27,28]. The myoblasts were also considered as nonprofessional APCs, with the capacity to drive the activation and proliferation of CD^4+^ T cell lines [29]. Muscle tissues are no longer passive bystanders but active participants in the immune response [30]. Analysis of the effect of adjuvant on local tissues can elucidate its mechanism more effectively and accord with its clinical application.

Here, the mechanisms of the adjuvant action of PD were explored using C2C12 myoblasts as an in vitro model, combined with an in vivo animal experiment. First, the rationality of using C2C12 cells as an in vitro model for studying the mechanism of PD was confirmed. It was further found that PD induced the inflammatory response in C2C12 cells via the Ca^2+^–c-jun N-terminal kinase (JNK)/p38 mitogen-activated protein kinase (MAPK)−caspase-1 pathway and caspase-1 mediated the inflammatory response and immune cell recruitment induced by PD at the injection site. Finally, it was successfully demonstrated that caspase-1-dependent pyroptosis mediates the adjuvant activity of platycodin D.

## 2. Materials and Methods

### 2.1. Materials

3-(4,5-Dimethylthiazol-2-yl)-2,5-diphenyltetrazolium bromide (MTT), concanavalin A (Con A), lipopolysaccharide (LPS), acridine orange (AO), ovalbumin (OVA), collagenase II, and rabbit anti-mouse IgG horseradish peroxidase (HRP)-conjugate (#A-9044) were purchased from Sigma-Aldrich, Saint Louis, MO, USA; fetal bovine serum (FBS) was obtained from Gibco, Grand Island, NY, USA; DMEM medium was obtained from Corning, Corning, NY, USA; goat anti-mouse IgG1 (#1070-05), IgG2a (#1080-05), and IgG2b (#1090-05) peroxidase conjugates were acquired from SouthernBiotech, Birmingham, AL, USA; mouse cytokine and chemokine detecting enzyme-linked immunosorbent assay (ELISA) kits were obtained from Boster Biological Technology co., Ltd., Wuhan, China; cyclic 3′,5′-adenosine monophosphate (cAMP) assay kits were acquired from Nanjing Jiancheng Bioengineering Institute, Nanjing, China; Fluo-3 AM was obtained from Dojindo Laboratories, Kumamoto, Japan; bicinchoninic acid (BCA) protein assay kit, reactive oxygen species (ROS) assay kit, enhanced chemiluminescence (ECL) kit, HRP-conjugated goat anti-rabbit (#A0208) and anti-mouse (#A0216) IgG (H+L), and radioimmunoprecipitation assay (RIPA) lysis buffer were acquired from Beyotime Biotech, Nantong, China; and the chromogenic end-point tachypleus amebocyte lysate (CE TAL) was obtained from Xiamen Bioendo Technology Co., Ltd., Xiamen, China. TRIzol reagent was purchased from Ambion, Austin, TX, USA; RevertAid™ M-MuLV reverse transcriptase was obtained from Fermentas, Amherst, NY, USA; diethylpyrocarbonate (DEPC), ribonuclease inhibitor, and oligo(dT)_18_ were acquired from Sangon Biotech, Shanghai, China; FastStart universal SYBR Green Master (ROX) was obtained from Roche Diagnostics, Indianapolis, IN, USA. NLRP3 inhibitor MCC950 (CP-456773, CRID3), caspase-1 inhibitor belnacasan (VX-765), caspase-3 inhibitor z-DEVD-fmk, receptor interacting serine/threonine kinase 1 (RIPK1) inhibitor necrostatin-1 (Nec-1), Ca^2+^ chelator BAPTA-AM, extracellular signal-regulated kinase (ERK) 1/2 inhibitor PD98059, JNK inhibitor SP600125, and p38 MAPK inhibitor SB203580 were acquired from Selleck Chemicals, Houston, TX, USA; caspase-1 inhibitor Ac-YVAD-CMK was obtained from Absin, Shanghai, China; pan-caspase inhibitor Z-VAD-FMK was obtained from TargetMol, Boston, MA, USA; the Annexin V-fluorescein isothiocyanate (FITC)/propidium iodide (PI) Apoptosis Kit was acquired from MultiSciences, Hangzhou, China; the phosphatase inhibitor cocktail and protease inhibitor cocktail were acquired from Bimake, Houston, TX, USA; rabbit anti-mouse IL-1β (D4T2D, #12426S), microtubule associated protein 1 light chain 3 alpha/beta (MAP1LC3A/B, LC3A/B, #4108), anti-rabbit p44/42 MAPK (ERK1/2, 137F5, #4695), phospho-p44/42 MAPK (ERK1/2, Thr202/Tyr204, D13.14.4E, #4370), p38 MAPK (D13E1, #8690), phospho-p38 MAPK (Thr180/Tyr182, D3F9, #4511), SAPK/JNK (#9252), phospho-SAPK/JNK (Thr183/Tyr185, 81E11, #4668), and phospho-nuclear factor κB (NF-κB) p65 (S536, 93HE, #3033S) mAbs were obtained from Cell Signaling Technology, Danvers, MA, USA; rabbit anti-mouse NF-κB p65 (C-20, #sc-372) and mouse anti-mouse caspase-1 (14F468, #sc-56036) mAbs were acquired from Santa Cruz Biotechnology, Santa Cruz, CA, USA; anti-mouse TATA-box binding protein (TBP) mAb (66166-1-Ig) was obtained from Proteintech, Chicago, IL, USA; and blue plus IV protein marker (DM131) was obtained from TransGen Biotech, Beijing, China. Anti-mouse CD16/CD32 purified (FcR Block), lymphocyte antigen 6 complex, locus G (Ly-6G) (Gr1)–PE-Cy5 (RB6-8C5, #15-5931-81), Ly-6C–APC (HK 1.4, #17-5932-82), CD11c–PE (N418, #12-0114-81), CD3e–PE-Cy5 (145-2C11, #15-0031-81), adhesion G protein-coupled receptor E1 (F4/80)–APC (BM8, #17-4801-82), CD117 (c-Kit)–PE-Cy5 (2B8, #15-1171-81), and Fc epsilon Receptor 1 alpha (FceR1)–APC (42795, #17-5898-82) mAbs were acquired from eBioscience, San Diego, CA, USA; CD45R–PE (RA3-6B2, #103208) was acquired from BioLegend, San Diego, CA, USA; sialic acid binding Ig-like lectin F (Siglec-F)–PE (E50-2440, #552126) was obtained from BD Pharmingen, San Diego, CA, USA; Alexa Fluor 488-conjugated ovalbumin was acquired from Invitrogen, Carlsbad, CA, USA; DNase I (#10530400) was obtained from Roche, Basel, Switzerland; and the SurePrint G3 8 × 60 K mouse gene expression microarray was provided by Agilent Technologies, Santa Clara, CA, USA.

PD (C_57_H_92_O_28_, MW: 1225.34) was isolated and prepared from the roots of *P. grandiflorum* according to a previously published method [11]. The purity of PD was determined to be more than 99% by high-performance liquid chromatography (HPLC). The endotoxin level in the PD solution of 10 mg/mL was measured to be considerably lower than 0.5 endotoxin units (EU)/mL by a CE TAL assay, indicating that the PD used in this study could be excluded from endotoxin contamination.

### 2.2. Cell Culture and Stimulation

The mouse C2C12 myoblast cell line was purchased from the cell bank of the Shanghai Branch of the Chinese Academy of Sciences, Shanghai, China, and maintained in a 5% CO_2_ atmosphere in DMEM medium, supplemented with 10% FBS, 100 μg/mL streptomycin, and 100 U/mL penicillin. The C2C12 cells were authenticated by short tandem repeat profiling prior to use and were tested to be negative for mycoplasma contamination. For all experiments, cells were plated one day prior to stimulation and were stimulated with PD at the various concentrations for the indicated time. Then, the cells or culture supernatants were collected for the MTT assay, fluorescence microscopy, flow cytometry, enzyme-linked immunosorbent assay (ELISA), real-time quantitative polymerase chain reaction (RT-qPCR), and Western blot analysis.

### 2.3. Mice

Female BALB/c mice aged 4–6 weeks were purchased from the Shanghai Experimental Animal Center of the Chinese Academy of Sciences (certificate no. SCXK 2007-0005), Shanghai, China. All experiments were in compliance with the People’s Republic of China’s legislation on the use and care of laboratory animals and followed the guidelines established by the Institute of Laboratory Animals of Zhejiang University, and approved by the University Animal Experimental Committee.

### 2.4. Injections

Mice were injected in the quadricep muscles on one leg with 50 μL of phosphate-buffered saline (PBS) as a control, and on the contralateral leg with 50 μg PD dissolved in 50 μL of PBS. Mice were anesthetized with 10% chloral hydrate and then sacrificed at the indicated time points, and then the quadricep muscle tissues were harvested from four mice per group. For the inhibition assay, mice were pre-injected intramuscularly (*i.m.*) with z-VAD-fmk or Ac-YVAD-CMK at the dose of 1 μg/g of body weight in 25 μL PBS 1 h before the intramuscular injection of PD (50 μg) dissolved in 25 μL of PBS. For ELISA, the muscle tissues were homogenized using TissueRuptor II handheld homogenizer (QIAGEN, Dusseldorf, Germany) in 1 mL PBS with a protease inhibitor cocktail. The supernatants were collected by centrifugation at 12,000 rpm for 10 min at 4 °C. The protein concentrations in the supernatants were detected by the BCA method using bovine serum albumin (BSA) as a standard. For the RT-qPCR, the muscle tissues were homogenized with 1 mL TRIzol reagent using TissueRuptor II handheld homogenizer [31].

### 2.5. Histological Observation

Mice were injected into the quadricep muscles of a unilateral leg with 50 μg PD dissolved in 50 μL of PBS. The quadricep muscle tissues were collected from four mice per group at 0, 0.5, 1, 2, and 4 h after PD injection, and then fixed with 4% paraformaldehyde, dehydrated through a series of graded ethanol, hyalinized with xylene, embedded in paraffin, and sectioned at 5-μm thicknesses. Microsections were stained with hematoxylin and eosin (H&E). The histological changes induced by PD were observed on an Olympus CKX53 microscope (Olympus, Tokyo, Japan) and compared with the untreated control group. A subjective histopathology score was recorded by an independent observer blinded to the nature of the specimens.

The number of inflammatory cells was scored from 0 to 4 [32]: 0, no infiltration; 1, mild, 5 to 25 inflammatory cells per high-power field (HPF; 40 × objective and 10 × ocular); 2, moderate, 26 to 50 inflammatory cells per HPF; and 3, severe, more than 50 inflammatory cells per HPF. The inflammatory cells were counted on four randomly selected HPF in each section [33], and the inflammation score was an average of the 4 selected HPF.

The degree of myonecrosis was scored as follows, based on an assessment of 4 fields at 200 × magnification in each section: 0, no necrotic fibers; 1, mild, <10% necrotic fibers; 2, moderate, 10% to 50% necrotic fibers; and 3, severe, >50% of necrotic fibers [32]. For the evaluation of fiber necrosis, the swollen, eosinophilic, vacuolated, and fragmented fibers were counted. The myonecrosis score was an average of the 4 selected fields.

Refer to the scoring criteria for myoedema [34], a modified scoring system was designed as follows, based on the assessment of 4 fields at 200 × magnification in each section: 0, no muscle edema; 1, mild, <10% edema of muscle bundles; 2, moderate, 10% to 50% edema of muscle bundles; and 3, severe, >50% edema of muscle bundles. The myoedema score was an average of the 4 selected fields.

The total muscle histopathology score for each section was a sum of the myoedema, myonecrosis, and inflammation scores, and was used to quantify muscle tissue damage.

### 2.6. Cell Viability Assay

The C2C12 cells were seeded at 1 × 10^4^ cell/well in a 96-well plate and incubated at 37 °C in a humidified atmosphere with 5% CO_2_. After 24 h, the various concentrations of PD were added into each well and these cells were incubated at 37 °C for 4, 8, 12, and 24 h, respectively. Each concentration was repeated for four wells. Three hours before the end, the cell proliferation was detected using MTT assay as previously described [35].

### 2.7. Fluorescence Microscopy

The C2C12 cells were seeded at 1 × 10^5^ cells/well into 24-well plates and then incubated at 37 °C in a humidified atmosphere with 5% CO_2_. After 24 h, the cells were treated with PD (25 μM) for 0, 1, or 24 h. After washed twice with PBS, cells were stained with 40 μL AO solution (50 μg/mL) for 10 min, and then visualized by fluorescence microscope (Olympus, Tokyo, Japan) with 488 nm stimulation and 500–520 nm emission [36].

### 2.8. Annexin V-FITC/PI Staining

The C2C12 cells were seeded at 1 × 10^5^ cells/well into 24-well plates and then cultured at 37 °C for 24 h in a humidified atmosphere with 5% CO_2_. After treatment with PD (25 μM) for 0.5, 1, 2, 4, or 6 h, the cells were harvested, washed twice with PBS, and then stained with the Annexin V-FITC/PI Apoptosis Kit according to the instructions. The analysis was performed on the FACSCalibur flow cytometer (BD Biosciences, San Jose, CA, USA) using FlowJo software (v10, BD Life Sciences).

### 2.9. Intracellular Free Calcium Detection

The intracellular free Ca^2+^ levels were measured by flow cytometry using the fluorescent dye Fluo-3 AM [37]. The C2C12 cells were seeded at 1 × 10^5^ cells/well into 24-well plates and then cultured at 37 °C for 24 h in a humidified atmosphere with 5% CO_2_. After being exposed to PD at 25 μM for different times, the C2C12 cells were incubated in 4 μM Fluo-3 AM at 37 °C for 30 min away from light. The harvested cells were washed twice with Hank’s Balanced Salt Solution (HBSS) and then detected for the mean fluorescence intensity (MFI) using a FACSCalibur flow cytometer (BD Biosciences, San Jose, CA, USA) at the excitation wavelength of 488 nm.

### 2.10. ROS Detection

The intracellular ROS levels were detected using the ROS assay kit [37]. The C2C12 cells were seeded at 1 × 10^5^ cells/well into 24-well plates and then cultured at 37 °C for 24 h in a humidified atmosphere with 5% CO_2_. After treatment with PD at 25 μM for 0, 30, 45, 60, and 120 min, the C2C12 cells were incubated with 10 μM 2′,7′-dichlorofluorescein diacetate (DCFH-DA) at 37 °C for 30 min, and then were washed three times with PBS containing 2% FBS. The MFI was determined by FACSCalibur flow cytometer (BD Biosciences, San Jose, CA, USA).

### 2.11. cAMP Analysis

The C2C12 cells were seeded at 1 × 10^5^ cells/well into 24-well plates and then cultured at 37 °C in a humidified atmosphere with 5% CO_2_. After incubation with PD at 25 μM for 0, 0.5, 1, 2, and 4 h, the cells were collected and lysed by an ultrasonic cell disruptor (BRANSON, Danbury, CT, USA) on pulse mode (on 10 s, off 30 s, 30% amplitude, 3 min). The supernatants were collected by centrifugation at 12000 rpm for 10 min at 4°C. The protein concentrations in the supernatants were detected by the BCA method using BSA as a standard. The concentrations of cAMP were measured using commercial kits according to the instructions. The data were standardized with protein concentrations.

### 2.12. Cytokine and Chemokine Analysis

For the C2C12 cells, 1 × 10^5^ cells were seeded in 24-well plates and allowed to rest for 24 h. The culture supernatants of the C2C12 cells after PD stimulation were collected. For the muscle tissue, the supernatants of the mouse muscle tissue homogenate were prepared according to the method mentioned above in “2.4. Injections”. The levels of cytokines (IL-1β, IL-6, IL-10, and interferon (IFN)-γ) and chemokines (CCL3 and CXCL2) in the supernatants of the cell culture and mouse muscle tissue homogenate were detected using commercial ELISA kits as previously described [35]. For the quadricep muscle tissues, the results were standardized with a protein concentration, and the values were expressed as pg/mg protein.

### 2.13. RT-qPCR

For the C2C12 cells, 1 × 10^5^ cells were seeded in 24-well plates and stimulated with or without PD at 25 μM. In the inhibition assay, 1 × 10^5^ cells were pretreated with or without the indicated inhibitors before PD stimulation. For the muscle tissue, the homogenate was prepared according to the method mentioned above in “2.4. Injections”. The total RNA was isolated with TRIzol reagent and reverse transcription was performed as previously [38]. The PCR was performed on an Applied Biosystems™ 7500 Real-Time PCR Systems (ABI Life Technologies, Foster, CA, USA) using FastStart Universal SYBR Green Master (Rox). The PCR cycling was performed as follows: initial denaturation at 95 °C for 10 min followed by 40 cycles of denaturation at 95 °C for 10 s, and annealing at 60 °C for 1 min. The specific primers for RT-qPCR were synthesized by Sangon Biotech Co., Ltd. (Shanghai, China) and the sequences were listed in Table S1. Primer amplification efficiency and specificity were verified for each set of primers. Glyceraldehyde-3-phosphate dehydrogenase (*Gapdh*) was used as an endogenous control. The mRNA expression levels of the tested genes relative to *Gapdh* were determined using the 2^-ΔΔCt^ method and shown as fold induction.

### 2.14. Western Blotting

The C2C12 cells were seeded at 1 × 10^6^ cells into a 6-cm dish and then incubated at 37 °C for 24 h in a humidified atmosphere with 5% CO_2_. After being treated with PD for various times, the C2C12 cells were washed twice with cold PBS and lysed with RIPA lysis buffer. The contents of the protein were measured with the BCA protein assay kit, using BSA as a standard. The denatured proteins were separated on 10–12% SDS-polyacrylamide gel electrophoresis (SDS-PAGE) and transferred to a polyvinylidene difluoride (PVDF) membrane. After blocking the membrane with 5% skim milk in Tris-buffered saline containing 0.1% Tween-20 (TTBS) for 1 h at 37 °C, the blot was incubated with anti-mouse TBP, caspase-1, IL-1β, anti-rabbit β-actin, LC3A/B, JNK, P-JNK, p38 MAPK, P-p38 MAPK, ERK1/2, P-ERK1/2, NF-κB p65, or P-NF-κB p65 mAbs overnight at 4 °C. Subsequently, the membranes were washed with TTBS and incubated with HRP-conjugated goat anti-mouse or anti-rabbit IgG for 1 h. After washing the membrane with TTBS three times, the signal was visualized with ECL on the LiCor C-DiGit Blot scanner using Image Studio Lite software (v5.0.60505, LI-COR Biosciences, Lincoln, NE) [37].

### 2.15. Microarray Analysis

Total RNA was further purified with the RNeasy^®^ Mini Kit (Qiagen, Nasdaq, NY, USA). Fluorescent complementary RNA (cRNA) was generated by Agilent’s Low Input Quick Amp Labeling Kit (Agilent Technologies, Santa Clara, CA, USA) and purified with the RNeasy^®^ Mini Kit (Qiagen, Nasdaq, NY, USA). The integrity of the input template RNA and labeled cRNA was determined on the NanoDrop UV-VIS spectrophotometer and the Agilent 2100 Bioanalyzer using the RNA 6000 Nano LabChip kit. RNA labeling and hybridization were performed according to the manufacture’s protocol. Hybridized microarrays were scanned with the Agilent C scanner using Agilent’s Scan Control software, version A.8.4.1. The features were extracted with Feature Extraction software. Data preprocessing and differential expression analysis were conducted using R software. The data were normalized using the quantile method (GeneSpring 12.0). Normalized expression data were subjected to log_2_ transformation. *p*-value < 0.05 and fold change > 2 were considered as a significant difference compared with untreated samples calculated on the three replicates [39]. A volcano plot was generated with the average fold change and *p*-values using a drawing tool on http://sangerbox.com/ (accessed on 12 March 2019). A Venn diagram was produced using http://bioinformatics.psb.ugent.be/webtools/Venn/ (accessed on 11 January 2019). K-means clustering analysis was performed to profile the gene expression pattern with MultiExperiment Viewer (v4.6.0, available online: https://mev.tm4.org, accessed on 12 March 2019) [40]. The Gene Ontology (GO) and Kyoto Encyclopedia of Genes and Genomes (KEGG) analyses were performed for the functions and pathways of the differentially expressed genes (DEGs) using Metascape Bioinformatics Resources (http://metascape.org/gp/index.html#/main/step1, accessed on 12 March 2019).

### 2.16. Inhibition Assay

For the in vitro analysis, after incubation with z-VAD-fmk (pan-caspase inhibitor, 50 μM), z-DEVD-fmk (caspase-3 inhibitor, 20 μM), VX-765 (caspase-1 inhibitor, 50 μM), Ac-YVAD-CMK (caspase-1 inhibitor, 25 μM), Nec-1 (RIPK1 inhibitor, 50 μM), BAPTA-AM (Ca^2+^ chelator, 10 μM), SP600125 (JNK inhibitor, 10 μM), or SB203580 (p38 inhibitor, 20 μM) for 0.5, 1, or 2 h, the C2C12 cells were stimulated with PD (25 μM) for 1 or 4 h. The cells and supernatants were collected for the Annexin V-FITC/PI staining, mRNA, and protein expression levels by flow cytometry, RT-qPCR, and Western blotting, respectively.

### 2.17. Immune Cell Recruitment into Muscles

Age- and body weight-matched BALB/c mice were divided into groups, each consisting of four mice. Groups of mice were injected with 25 μL PBS, MCC950 (3 mg/kg of body weight, 30 min), or Ac-YVAD-CMK (1 mg/kg of body weight, 1 h) per quadricep on two legs. After the indicated time, mice were injected *i.m.* with 25 μL/quadricep muscle of OVA-AF488 (10 μg), alone or in the presence of PD (50 μg). Twenty-four hours later, the quadricep muscles were harvested from all four mice per group, cut into small pieces, and then digested with 0.05% collagenase II and DNase I (10 mg/mL) in PBS at 37 °C for 30 min. After centrifugation, the pelleted cells were suspended in DMEM, and filtered through a 70 μm nylon mesh to obtain a cell suspension. The cell suspension was centrifuged and washed with PAB (1% bovine serum albumin and 0.1% sodium azide in PBS). The cells were blocked with 1 μg of purified anti-mouse CD16/CD32 antibody for 10 min to inhibit nonspecific staining, and then stained at room temperature for 30 min with combinations of anti-mouse Ly-6G–PE-Cy5, Ly-6C–APC, and CD11c–PE, or CD3e–PE-Cy5, F4/80–APC, and CD45R–PE, or CD117–PE-Cy5, FceR1–APC, and Siglec-F–PE [41]. The stained cells were analyzed on the FACSCalibur flow cytometer (BD Biosciences, San Jose, CA, USA) using FlowJo software (v10, BD Life Sciences).

### 2.18. Adjuvant Activity Assessment

Age- and body weight-matched BALB/c mice were divided into groups, each consisting of five mice. The animals were immunized *i.m.* with OVA (10 μg), alone or in combination with PD (50 μg) in 25 μL of PBS on Day 1. A boosting injection was given 2 weeks later. PBS-treated animals were included as controls. To inhibit the caspases or caspase-1 in the local tissues, mice were injected *i.m*. with z-VAD-fmk or Ac-YVAD-CMK at the dose of 1 μg/g of body weight in 25 μL PBS 60 min before the start of immunization (on days 1 and 15). Sera and splenocytes were collected 2 weeks after the second immunization. Serum OVA-specific IgG antibody and its isotype titers in OVA-immunized mice were determined by an indirect ELISA [10,11]. Splenocytes (5 × 10^5^ cells/well) were seeded into a 96-well cell culture plate, and then incubated with Con A (5 μg/mL), LPS (10 μg/mL), OVA (20 μg/mL), or RPMI for 44 h at 37 °C and 5% CO_2_. Splenocyte proliferation was detected by the MTT method [10,11]. Splenocytes (1 × 10^6^ cells/well) and K562 cells (2 × 10^4^ cells/well) were seeded in a 96-well U-bottom microtiter plate in RPMI complete medium and then incubated for 20 h at 37 °C in 5% CO_2_. The activities of natural killer (NK) cells in splenocytes against human leukemia K562 cells were assured by MTT assay [10]. Splenocytes (5.0 × 10^6^ cells/well) were seeded into a 24-well cell culture plate, and then stimulated with OVA (20 μg/mL) for 72 h at 37 °C and 5% CO_2_. The supernatants were harvested for the detection of IFN-γ and IL-10 by ELISA kits [35]. Splenocytes (5.0 × 10^6^ cells/well) were seeded into a 24-well cell culture plate, and then stimulated with OVA (20 μg/mL) for 18 h at 37 °C and 5% CO_2_. The cells were collected for measuring the mRNA expression levels of *Il-2*, *Il-4*, *Il-10*, and *Ifn-γ* by RT-qPCR [38].

### 2.19. Statistical Analysis

Data were presented as mean ± SD and examined for their statistical significance of difference with Analysis of Variance (ANOVA) and Student’s *t*-test. The *p*-values of less than 0.05 were considered to be statistically significant. The calculations and graphs were performed using GraphPad Prism 8.0 software (GraphPad Software, San Diego, CA, USA).

## 3. Results

### 3.1. PD Led to Tissue Damage and Inflammatory Response in Mouse Quadricep Muscles

**Figure 1:**
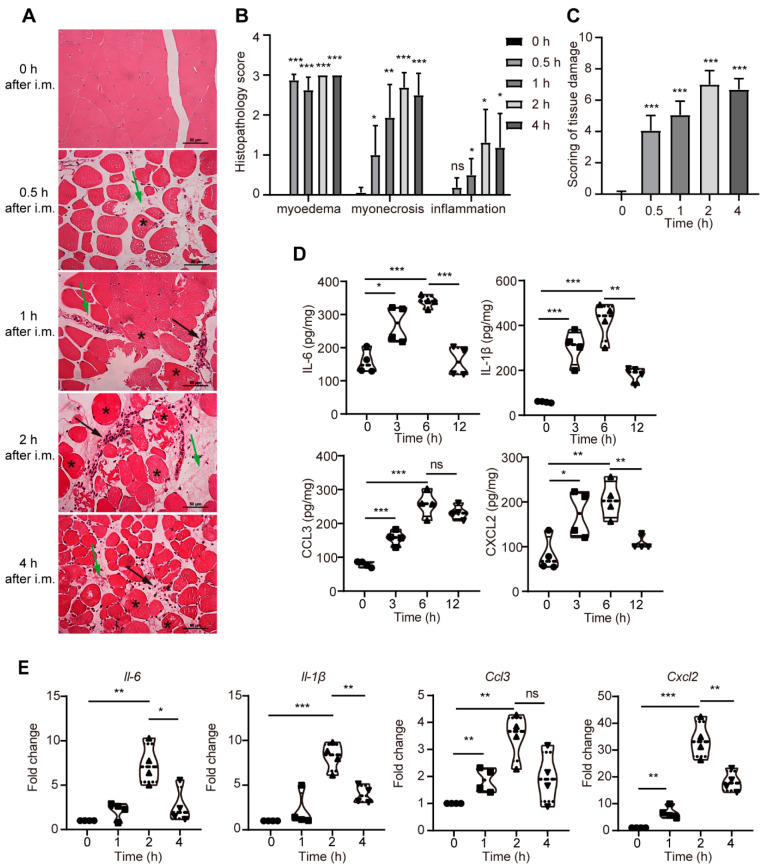
The intramuscular injection of PD led to tissue damage and inflammatory response in mouse quadricep muscles. (**A**) The quadricep muscle sections were stained using H&E. The light photomicrographs shown were representative of quadricep muscle sections from four mice per group. Edema (green arrows); inflammation, the muscle fibers are surrounded by inflammatory cells, mainly neutrophils (black arrows); and myonecrosis (asterisk). Scale bars: 50 μm. (**B**) Histopathology score of myoedema, myonecrosis, and inflammation. (**C**) Quantification of muscle tissue damage by the total muscle histopathology score. (**D**) The levels of IL-6, IL-1β, CCL3, and CXCL2 in quadricep muscles injected *i.m.* with 50 μg PD by ELISA. (**E**) The gene expression levels of *Il-6*, *Il-1β*, *Ccl3*, and *Cxcl2* in quadricep muscles injected *i.m.* with 50 μg PD by RT-qPCR. Data were presented as mean ± SD (*n* = 3, the number of replicates, the same below). (*) *p* < 0.05, (**) *p* < 0.01, (***) *p* < 0.001, ns not significant.

Some TLR-independent adjuvants, such as aluminum compounds (Alum), MF59, and Matrix-M™, have been shown to function by evoking a cytokine and chemokine microenvironment at the vaccination site [42]. To evaluate the degree of injury to the quadricep muscles after intramuscular injection of PD, the histopathological changes were observed by H&E staining [43]. Compared to the normal control, PD resulted in significant injury, dominated by the myoedema (green arrows), myonecrosis (asterisks), and inflammation (black arrows) (Figure 1A). The myoedema and myonecrosis scores were significantly higher 0.5 h after injection with PD, while the inflammation score was significantly increased at 1 h (Figure 1B). Quantitative analysis of overall tissue damage showed that the score had significantly increased at 0.5 h and slightly decreased at 4 h compared with the normal group (Figure 1C).

The cytokines and chemokines play a key role in inducing the recruitment of various immune cells at the injection site. The effects of PD on the level of the cytokines (IL-1β and IL-6) and chemokines (CCL3 and CXCL2) at the injection site were examined by ELISA. As shown in Figure 1D, the levels of IL-6, IL-1β, CCL3, and CXCL2 in the PD-injected quadricep muscles rocketed and reached a peak at 6 h, whereas there was a notable reduction in their levels after 12 h. RT-qPCR analysis showed that PD drastically upregulated the mRNA expression levels of *Il-6*, *Il-1β*, *Ccl3*, and *Cxcl2* in the quadricep muscles. The mRNA expression levels of these proinflammatory factors were upregulated at 1 h, peaked at 2 h, and gradually declined at 4 h following injection (Figure 1E). These findings indicated that the intramuscular injection of PD resulted in a rapid and transient inflammatory response at the injection site.

### 3.2. PD Induced Transient Cytotoxicity and Inflammatory Response in C2C12 Cells

**Figure 2:**
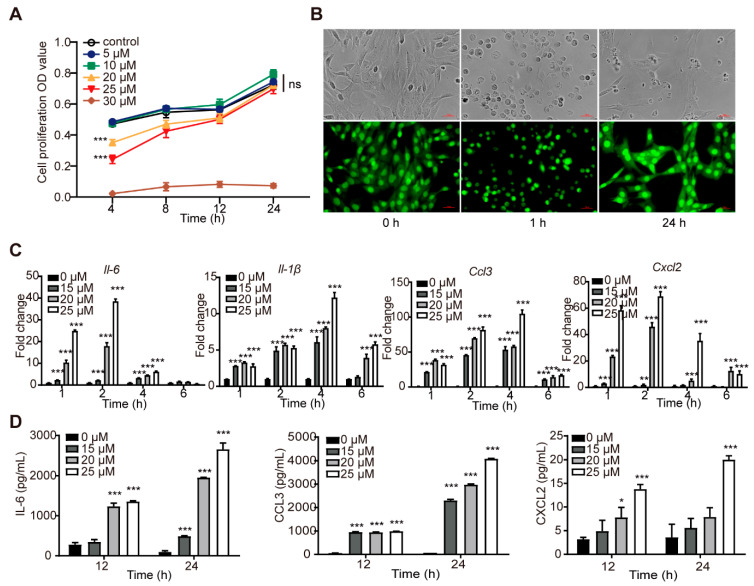
PD-induced transient cytotoxicity and inflammatory response in C2C12 cells. (**A**) Cell viability assay by MTT method. (**B**) Microscopic pictures of optical cell morphology showing the cytotoxicity of PD (25 μM) towards C2C12 cells by AO staining. (**C**) The gene expression of *Il-6*, *Il-1β*, *Ccl3*, and *Cxcl2* in C2C12 cells by RT-qPCR. (**D**) The levels of IL-6, CCL3, and CXCL2 in the culture supernatants of C2C12 cells by ELISA. Data were presented as mean ± SD (*n* = 3). (*) *p* < 0.05, (**) *p* < 0.01, (***) *p* < 0.001, ns not significant.

The effects of PD on the growth of the C2C12 cells were measured using the MTT method. PD showed significant concentration-dependent cytotoxicity towards the C2C12 cells at the concentration of more than 10 μM for 4 h, with the IC_50_ value being 23.8 μM. There were, however, no significant differences in the OD values among the C2C12 cells treated with PD at 0–25 μM after 24 h (Figure 2A). Morphological observation revealed that the C2C12 cells were contracted and lytic 1 h after treatment with PD at 25 μM (Figure 2B). However, PD-treated cells were restored to the long fusiform shape and even had a stronger green fluorescence after 24 h. These results indicated that PD-induced cytotoxicity towards the C2C12 cells was transient.

The mRNA expression levels of proinflammatory cytokines and chemokines in the C2C12 cells treated with PD at the various concentrations for different times were detected by RT-qPCR. PD significantly upregulated the mRNA expression levels of *Il-6*, *Il-1β*, *Ccl3*, and *Cxcl2* in the C2C12 cells in a time- and concentration-dependent manner (Figure 2C). The mRNA expression levels of these proinflammatory factors peaked at 2−4 h after PD treatment, and then quickly descended. The effects of PD on the secretion of proinflammatory cytokines and chemokines from the C2C12 cells were also detected using ELISA. As shown in Figure 2D, PD concentration- and time-dependently promoted remarkably the secretion of IL-6, CCL3, and CXCL2 from the C2C12 cells.

PD exhibited the similar in vivo and in vitro cytotoxicity characterized by cell contraction and lysis, accompanied by upregulation of cytokines and chemokines at the gene and protein levels.

### 3.3. PD Induced Similar Gene Expression Profiles in C2C12 Cells and Mouse Quadricep Muscles

**Figure 3:**
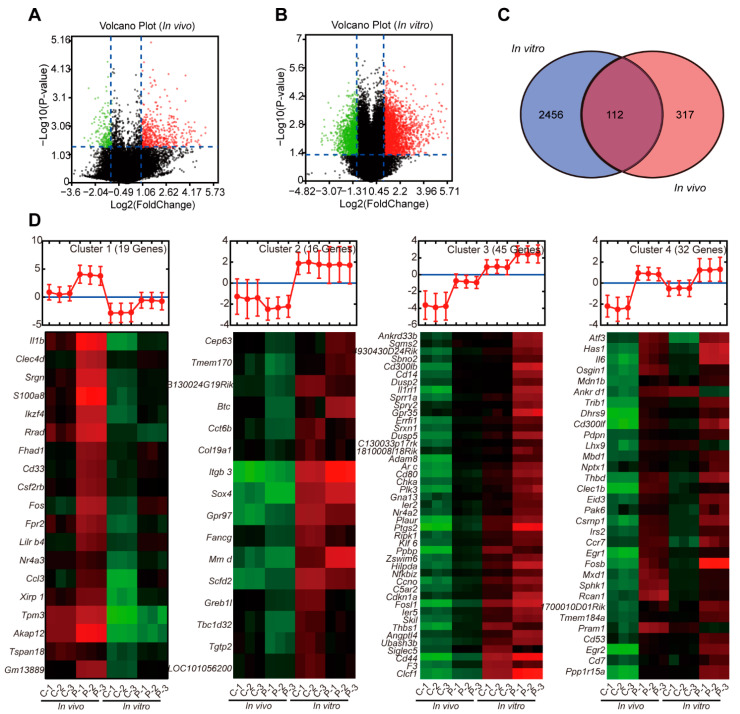
PD induced similar gene expression profiles in C2C12 cells and mouse quadricep muscles. C2C12 cells were stimulated with or without PD at 25 μM. Mice were *i.m.* injected with 50 μg PD at the quadricep muscles. The cells (for 4 h) and muscle tissues (2 h) were well collected and subjected to microarray analysis. (**A**,**B**) Volcano plots of gene expression in C2C12 cells (**A**) and quadricep muscles (**B**). (**C**) The Venn diagram showed the distribution of DEGs in PD-treated C2C12 cells and quadricep muscles. (**D**) Expression pattern analysis of 112 coregulated DEGs by *k*-means clustering with MultiExperiment Viewer.

To further comprehensively evaluate the feasibility of C2C12 cells as an in vitro model for studying adjuvant mechanism, the C2C12 cells and mouse quadricep muscles treated with PD were subjected to a SurePrint G3 8 × 60 K mouse gene expression microarray. PD induced 3410 DEGs in the C2C12 cells at 25 μM for 4 h. Among them, 1921 genes were upregulated and 1489 genes were downregulated (Figure 3A). Similarly, PD resulted in 650 DEGs in the quadricep muscles 2 h after intramuscular injection, among which 438 genes were upregulated and 212 genes were downregulated (Figure 3B). The Venn diagram showed that 112 common DEGs were regulated in the C2C12 cells and mouse quadricep muscles by PD (Figure 3C). *K*-means cluster analysis showed that, although the basal expression levels of these coregulated DEGs in the C2C12 cells and quadricep muscles were inconsistent, the average expression level of the second cluster showed a small downward trend and the other clusters showed a similar upward trend, indicating an analogous expression pattern in vitro and in vivo (Figure 3D).

**Figure 4:**
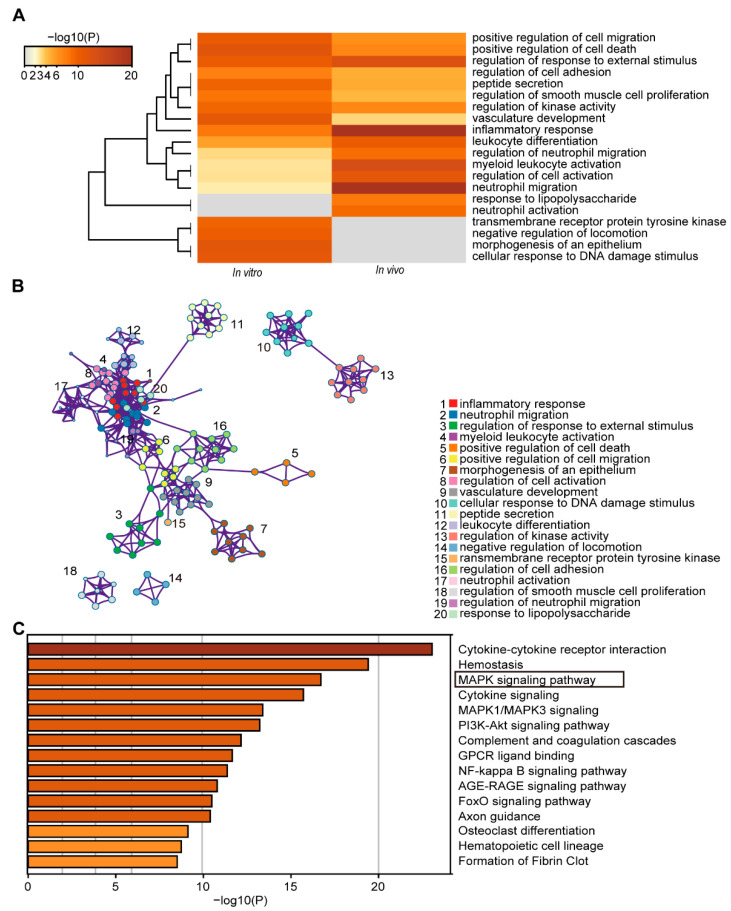
The GO analysis of PD-induced DEGs in C2C12 cells and mouse quadricep muscles. (**A**) GO biological processes of DEGs using Metascape. (**B**) Network of GO biological processes of DEGs. Each term was represented by a circle node, where its size was proportional to the number of genes falling into that term; nodes of the same color belonged to the same cluster, and an edge linked terms with a similarity score > 0.3; the thickness of the edge represented the similarity score. (**C**) KEGG enriched pathways of the DEGs in 14 coregulated clusters related to inflammation, cell death, and immunity, by Metascape.

The Gene Ontology (GO) analysis of PD-induced DEGs in the C2C12 cells and mouse quadricep muscles was performed to compare their biological process functions. Fourteen of the top 20 clusters were found to be coregulated and were all associated with inflammation, death, and immunity (Figure 4A), suggesting the similar biological processes of DEGs induced by PD in the C2C12 cells and quadricep muscles. Meanwhile, a network was generated for clarifying the relationship among the top 20 clusters using Metascape. Except for the individual clusters of ‘regulation of smooth muscle cell proliferation’ and ‘negative regulation of locomotion’, the other terms were interrelated and formed a biological process centered on death, inflammation, and immunity (Figure 4B), further confirming that PD could induce the cytotoxicity, inflammatory, and immune response in the C2C12 cells and mouse quadricep muscles.

Pathway enrichment analysis of the DEGs from the above 14 coregulated clusters was performed using Metascape, based on the Kyoto Encyclopedia of Genes and Genomes (KEGG) and Reactome Gene Sets databases to identify the pathways involved in inflammation, death, and immunity. The most significant pathways were ‘cytokine–cytokine receptor interaction’, ‘hemostasis’, and ‘MAPK signaling pathway’ (Figure 4C). The cluster represented by ‘cytokine–cytokine receptor interaction’ consisted of ‘cytokine signaling in immune system’ and ‘signaling by interleukins’. The cluster represented by ‘hemostasis’ contained ‘response to elevated cytosolic Ca^2+^’. The above results indicated that PD might regulate the expression of death, inflammation, and immune-related genes via the Ca^2+^−MAPK pathway.

### 3.4. Multiple Cell Death Pathways Were Involved in PD-Induced Cytotoxicity

**Figure 5:**
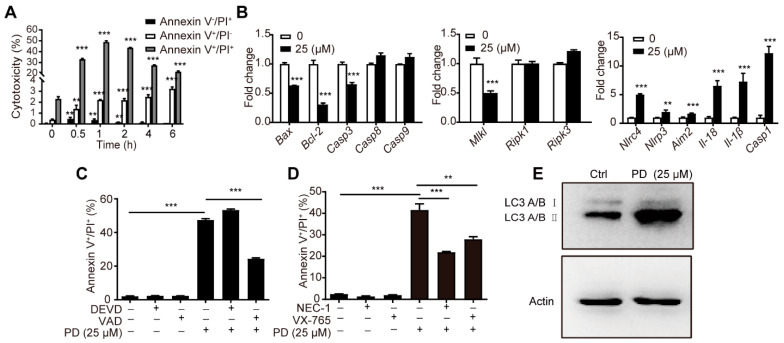
Multiple cell death pathways were involved in PD-induced cytotoxicity. (**A**) The proportion of Annexin V^+^/PI^-^ (early apoptotic), Annexin V^+^/PI^+^ (necrotic-like), and Annexin V^-^/PI^+^ (necrotic) in C2C12 cells after stimulation with PD (25 μM) at the indicated time. (**B**) The gene expression of biomarkers associated with cell apoptosis, necroptosis, and pyroptosis by RT-qPCR after stimulation with PD (25 μM) for 4 h. *Casp1/3/8/9*, caspase-1/3/8/9. (**C**,**D**) The proportion of Annexin V^+^/PI^+^ (necrotic-like) cells in C2C12 cells pretreated with indicated inhibitors before PD (25 μM) stimulation for 1 h. (**E**) C2C12 cells were stimulated with PD (25 μM) for 30 min, and the LC3A/B II protein levels were analyzed by immunoblotting. The figures shown were representative of three independent experiments. Data were presented as mean ± SD (*n* = 3). (**) *p* < 0.01, (***) *p* < 0.001, ns not significant.

The microarray analysis suggested that PD regulated the cell death pathway. Therefore, we first identified the specific death type induced by PD in the C2C12 cells. PD significantly increased the cell populations of Annexin V^+^/PI^+^ in a time-dependent manner (Figure 5A and Figure S1A), rather than Annexin V^+^/PI^-^ (early apoptosis) or Annexin V^-^/PI^+^ (necrosis), suggesting that PD-induced cell death is mainly a necrotic-like programmed cell death, characterized by changes in membrane permeability, such as secondary necrosis [44,45], a natural outcome of the complete apoptotic program, necroptosis [46,47], and pyroptosis [48], etc.

The effects of PD on the mRNA expression levels of molecular biomarkers associated with apoptosis, necroptosis, and pyroptosis were detected using RT-qPCR. PD significantly upregulated the mRNA expression levels of caspase-1, *Il-18*, *Il-1β*, NLR family CARD domain containing 4 (*Nlrc4*), *Nlrp3*, and absent in melanoma 2 (*Aim2*) involved in pyroptosis (Figure 5B). However, the mRNA expression levels of necroptosis-related *Ripk1*, *Ripk3*, and mixed lineage kinase domain like pseudokinase (*Mlkl*), as well as apoptosis-related *Bcl-2*, BCL2 associated X (*Bax*), caspase-3, -8, and -9 were unchanged or even downregulated (Figure 5B). Simultaneously, the effects of the pretreatment with specific inhibitors of pan-caspase (z-VAD-FMK, 50 μM), caspase-3 (z-DEVD-FMK, 20 μM), RIPK1 (Nec-1, 50 μM), and caspase-1 (VX-765, 50 μM) on the cell populations of Annexin V^+^/PI^+^ induced by PD were investigated using Annexin V-FITC/PI staining. The z-VAD-FMK, VX-765, and Nec-1 significantly decreased the Annexin V^+^/PI^+^ cells (Figure 5C,D and Figure S1B,C), while z-DEVD-FMK had no effect on the cytotoxicity of the PD-treated C2C12 cells (Figure 5C and Figure S1B), suggesting that pyroptosis and necroptosis may be involved in the PD-triggered cytotoxicity in the C2C12 cells, and apoptosis is not.

Physiological levels of autophagy promote cellular survival in response to a variety of stresses, while excessive activation of autophagy leads to autophagy-dependent cell death with plasma membrane rupture properties [49]. PD upregulated the protein levels of LC3A/B II in C2C12 cells (Figure 5E), suggesting that autophagy might be involved in PD-induced cytotoxicity. PD induced multipathway cell death in the C2C12 cells.

### 3.5. Ca^2+^−JNK/p38 MAPK−NLRP3 Inflammasome−Caspase-1 Pathway Was Essential for the Inflammatory Response in C2C12 Cells by PD

**Figure 6:**
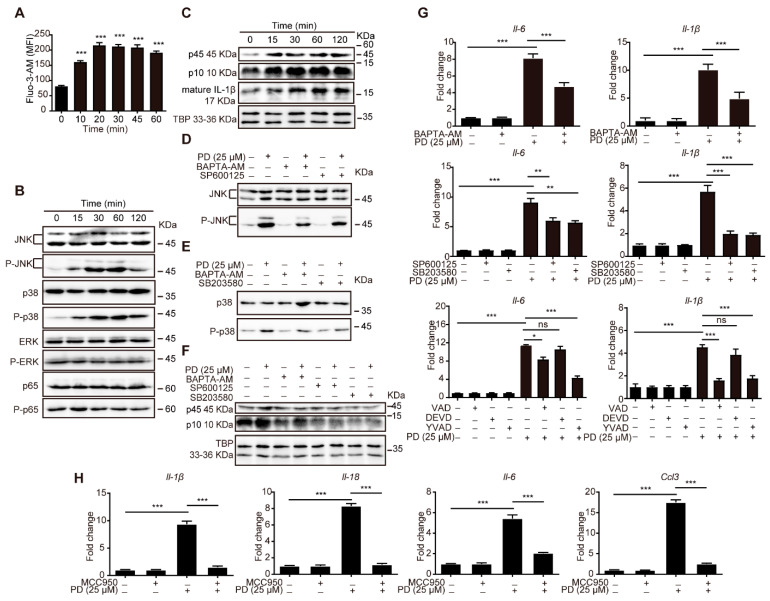
Ca^2+^−JNK/p38 MAPK−NLRP3 inflammasome–caspase-1 pathway was essential for the inflammatory response in C2C12 cells by PD. (**A**) The levels of intracellular free calcium in C2C12 cells treated with PD (25 μM) for 0–60 min by flow cytometry. (**B**) C2C12 cells were treated with PD (25 μM) for 0, 15, 30, 60, and 120 min, and the protein levels were detected by Western blotting. The figure shown was representative of three independent experiments. (**C**) C2C12 cells were stimulated with PD (25 μM) at the indicated time, and the activated caspase-1 and mature IL-1β protein levels were analyzed by immunoblotting. p45, pro-caspase-1; p10, activated caspase-1. The figures shown were representative of three independent experiments. (**D**–**F**) After pre-incubation with or without BAPTA-AM (10 μM, 30 min), SP600125 (10 μM, 1h), or SB203580 (20 μM, 1 h), C2C12 cells were treated with medium or PD (25 μM) for 1 h, and the protein levels of JNK/P-JNK (**D**), p38/P-p38 (**E**), and caspase-1 (**F**) were detected by Western blotting. The figure shown was representative of three independent experiments. (**G**) After pre-incubation with or without BAPTA-AM (10 μM, 30 min), SP600125 (10 μM, 1 h), SB203580 (20 μM, 1 h), or Ac-YVAD-CMK (25 μM, 2 h), C2C12 cells were treated with medium or PD (25 μM) for 4 h, and the gene expression levels of *Il-6* and *Il-1β* were detected by RT-qPCR. (**H**) C2C12 cells were pretreated with NLRP3 inhibitor (MCC950, 50 μM, 30 min) before PD (25 μM) stimulation for 4 h. The gene expression levels of *Il-1β*, *Il-18*, *Il-6*, prostaglandin-endoperoxide synthase 2 (*Ptgs2*), and *Ccl3* in C2C12 cells were determined by RT-qPCR. Data were presented as mean ± SD (*n* = 3). (*) *p* < 0.05, (**) *p* < 0.01, (***) *p* < 0.001, ns not significant.

The microarray analysis showed that PD potentially activates the Ca^2+^/MAPK pathway (Figure 4). The intracellular Ca^2+^ levels in C2C12 cells were first examined using a Fluo-3 AM Ca^2+^-sensitive fluorescent probe. PD induced a significant intracellular Ca^2+^ flux in the C2C12 cells in a concentration-dependent manner (Figure S2A). The Ca^2+^ levels in the C2C12 cells were elevated at 10 min and peaked at 20 min after PD stimulation (Figure 6A and Figure S2B).

MAPKs including ERK, JNK, and p38 MAPK play important roles in regulating cytokine release [50]. On the other hand, NF-κB is a pleiotropic regulator of many genes involved in immune response and regulates the expression of proinflammatory cytokines and chemokines [51]. PD significantly induced the phosphorylation of JNK and p38 MAPK in C2C12 cells from 15 min to 120 min. However, no significant differences were found in the phosphorylation of ERK and NF-κB p65 between the PD-treated and control C2C12 cells (Figure 6B). These results suggested that PD activated Ca^2+^−JNK/p38 pathways.

It is well known that caspase-1 is a key molecule in the classical pyroptosis pathway and has been reported to be regulated by Ca^2+^−MAPK signaling [52,53]. In addition, considering that PD induced the pyroptosis in the C2C12 cells (Figure 5B,C), we further validated the Ca^2+^−JNK/p38 MAPK−caspase-1 pathway.

PD significantly upregulated the protein expression levels of intracellular activated caspase-1 and mature IL-1β (Figure 6C and Figure S3). The Ca^2+^ chelator BAPTA-AM, JNK inhibitor SP600125, and p38 MAPK inhibitor SB203580 remarkably inhibited the upregulated phosphorylation of JNK and p38 MAPK (Figure 6D,E), and caspase-1 activation (Figure 6F) induced by PD in the C2C12 cells. Moreover, the pretreatment with SP600125, SB203580, and caspase-1 specific inhibitor Ac-YVAD-CMK also significantly suppressed the upregulated mRNA expression of *Il-6* and *Il-1β* in PD-treated C2C12 cells, whereas caspase-3 inhibitor z-DEVD-FMK failed to block (Figure 6G). These findings indicated that caspase-1 dependent pyroptosis was involved in the inflammatory response induced by PD in the C2C12 cells through the Ca^2+^−JNK/p38 MAPK−caspase-1 pathway.

Activation of caspase-1 is achieved through pro-caspase-1 shearing, which is mediated by a cytosolic multiprotein signaling platform called the inflammasome. NLRP3 is one of the key regulatory proteins in the formation of the inflammasome complex [54]. The NLRP3 inhibitor MCC950 significantly decreased the upregulated mRNA expression levels of *Il-1β*, *Il-18*, and other inflammatory cytokines such as *Il-6*, *Ptgs2*, and *Ccl3* (Figure 6H) induced by PD in the C2C12 cells. These results suggested that the NLRP3 inflammasome–caspase-1 pathway mediated the inflammatory response induced by PD in the C2C12 cells.

### 3.6. The NLRP3 Inflammasome-Caspase-1 Pathway Mediated the Inflammatory Response and Immune Cell Recruitment at the Injection Site Induced by PD

**Figure 7:**
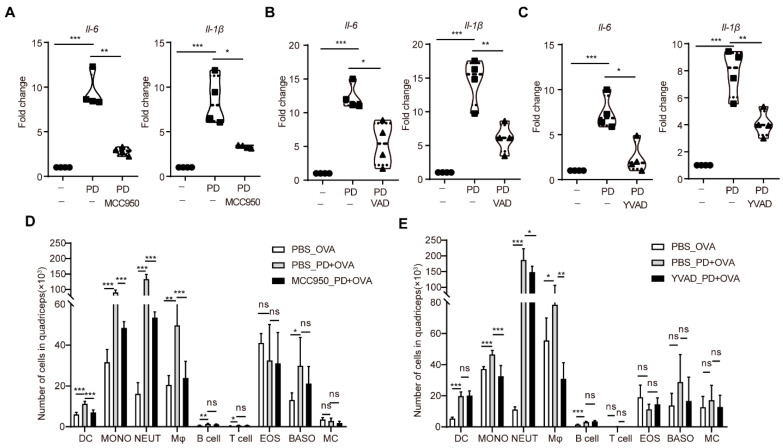
The NLRP3 inflammasome–caspase-1 pathway mediated the inflammatory response and immune cell recruitment induced by PD at the injection site. (**A**–**C**) Mice were injected *i.m.* with MCC950 ((**A**), 3 mg/kg, 30 min), z-VAD-FMK ((**B**), 1 mg/kg, 1 h), or Ac-YVAD-CMK ((**C**), 1 mg/kg, 1 h), and with PD (50 μg) in the quadricep muscles at the indicated time interval. After 2 h, the quadricep muscle tissues were collected and assayed for the gene expression levels of *Il-6* and *Il-1β* by RT-qPCR. (**D**,**E**) Mice were injected *i.m.* with MCC950 ((**D**), 3 mg/kg, 30 min) or Ac-YVAD-CMK ((**E**), 1 mg/kg, 1 h), and with PD (50 μg) in the quadricep muscles at the indicated time interval. After 24 h, the quadricep muscle tissues were collected and assayed for the number of various immune cells. Data were presented as mean ± SD (*n* = 3). (*) *p* < 0.05, (**) *p* < 0.01, (***) *p* < 0.001, ns not significant. DC, dendritic cell; MONO, monocyte; NEUT, neutrophil; Mφ, macrophage; EOS, eosinophil; BASO, basophil; MC, mast cell.

The recruitment of innate immune cells into the injection site is a critical function of adjuvants and a direct consequence of the local production of cytokines, as well as affecting the quality and magnitude of the immune response. The pretreatment of NLRP3 inhibitor MCC950, pan-caspase inhibitor z-VAD-FMK, and caspase-1 inhibitor Ac-YVAD-CMK significantly downregulated the mRNA expression levels of *Il-6* and *Il-1β* in PD-injected quadricep muscle tissues (Figure 7A–C), indicating that the blockade of NLRP3 and caspase-1 could inhibit the PD-induced local inflammatory response.

Meanwhile, we further analyzed the cell recruitment induced by PD into the injection site in the presence or absence of MCC950 or Ac-YVAD-CMK. After 24 h, muscle tissues were harvested and the number of dendritic cells (CD11c^+^Ly-6C^−^Ly6G^−^), neutrophils (CD11c^−^Ly-6C^+^Ly6G^high^), monocytes (CD11c^−^Ly6G^−^Ly6C^+^), macrophages (CD3^−^CD45R^−^F4/80^high^), T cells (CD3^+^CD45R^−^F4/80^−^), B cells (CD3^−^CD45R^+^F4/80^−^), eosinophils (SigiLecF^+^CD117^−^), basophils (SigLec F^−^FcER1^+^CD117^−^), and mast cells (SigLec F^−^CD117^+^FcER1^+^) were determined by flow cytometer. PD significantly induced the recruitment of dendritic cells, neutrophils, inflammatory monocytes, and macrophages into the injected quadricep muscles, compared with OVA-injected control mice (Figure 7D,E and Figure S4). However, MCC950 and Ac-YVAD-CMK significantly decreased the number of macrophages, neutrophils, and monocytes in the PD-injected quadricep muscles (Figure 7D,E). These results suggested that the NLRP3 inflammasome–caspase-1 pathway mediated the proinflammatory response and immune cell recruitment induced by PD at the injection site.

### 3.7. Caspase-1 Mediated the Adjuvant Activity of PD

**Figure 8:**
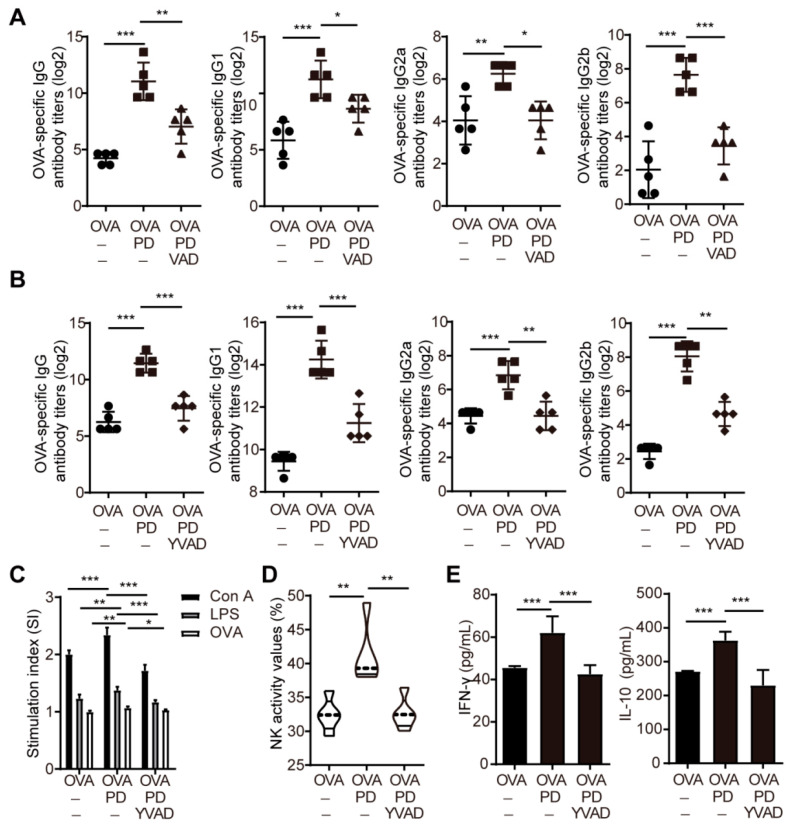
Caspase-1 mediated the adjuvant activity of PD. (**A**,**B**) Mice were injected *i.m.* with z-VAD-FMK (**A**) or Ac-YVAD-CMK (**B**) at the dose of 1 μg/g for 1 h before immunization. Sera were collected 2 weeks after the secondary immunization, and serum OVA-specific IgG, IgG1, IgG2a, and IgG2b antibodies were measured by an indirect ELISA. (**C**–**E**) Mice were injected i.m. with Ac-YVAD-CMK at the dose of 1 μg/g for 1 h before immunization. Splenocytes were prepared 2 weeks after the secondary immunization. Splenocyte proliferation (**C**) and NK cell activity (**D**) were measured by the MTT method. (**E**) The culture supernatants were measured for the levels of IFN-γ and IL-10 by ELISA. Data were presented as mean ± SD (*n* = 3). (*) *p* < 0.05, (**) *p* < 0.01, (***) *p* < 0.001, ns not significant.

An acute inflammatory response and strong recruitment of immune cells at the injection site promoted the antigen uptake and transport to draining lymph nodes, leading to overall strongly enhanced adaptive immune responses. Therefore, we further evaluated the role of caspase-1 in mediating the adjuvant activity of PD on the immune responses to OVA in mice. PD not only significantly enhanced the serum OVA-specific IgG, IgG1, IgG2a, and IgG2b antibody titers, but promoted splenocyte proliferation and NK cell activities, induced the production of IFN-γ and IL-10, as well as upregulated the mRNA expression levels of Th1 (*Il-2* and *Ifn-γ*) and Th2 (*Il-4* and *Il-10*) cytokines in OVA-stimulated splenocytes from the OVA-immunized mice (Figure 8A–E and Figure S5), which was consistent with the previous reports [10,11]. However, the pre-injection of z-VAD-FMK and Ac-YVAD-CMK into the quadricep muscles significantly decreased the serum OVA-specific IgG, IgG1, IgG2a, and IgG2b antibody titers in the mice immunized with OVA+ PD (Figure 8A,B). Moreover, the pre-injection of Ac-YVAD-CMK also significantly inhibited Con A-, LPS-, and OVA-stimulated splenocyte proliferation (Figure 8C), NK cell activities (Figure 8D), the production of IFN-γ and IL-10 (Figure 8E), and the mRNA expression levels of *Il-2*, *Il-4*, *Il-10*, and *Ifn-γ* (Figure S5) in OVA-stimulated splenocytes from the mice immunized with OVA+PD. These findings indicated that caspase-1 mediated the adjuvant activity of PD.

## 4. Discussion

The innate immune cells are activated through pattern recognition receptors (PRRs). However, this could hardly explain the immune responses to some adjuvants that remarkably activate the immune system but no PRRs have been identified yet. The ‘danger’ model suggests that immunity might be guided by danger-associated molecular patterns (DAMPs) released from the dying cells [55,56]. In addition to inducing the adaptive immune response, DAMPs released from dead cells induce an inflammatory response, and the time overlap reflects a mechanistic link between inflammatory response and adjuvant effects [57]. Alum has been shown to have cytotoxic effects, which could result in the release of host DNA into the cytoplasm and then influence its adjuvanticity [58,59]. QS-21 induced the death of macrophages and DCs in a caspase-1-, PYD and CARD domain containing (PYCARD, ASC)-, and NLRP3-independent manner at higher concentrations, which impacted its adjuvant effects [18]. Although the mechanisms of action of SBAs are being intensively investigated using immune cells [14,15,16], they are poorly elucidated [60].

The intramuscular injection is the most common vaccination route in the clinic. In view of the fact that muscle cells dominate in muscle tissues, in this study, C2C12 myoblasts were used as an in vitro model for exploring the mechanisms of action of SBAs. Adjuvants such as Alum, MF59 [61,62], AS03 [63], AS04 [64], and AJSAF (*Albizia julibrissin* saponin active fraction) [65] have been reported to induce the production of cytokines and chemokines at the injection site, recruit immune cells to the local tissues, and then load antigens to migrate to lymph nodes resulting in an enhanced adaptive immune response. PD was also found to induce a transient cytotoxicity and inflammatory response both in the C2C12 cells and in the mouse quadricep muscles. Moreover, there were similar GO biological processes and KEGG pathways of DEGs in the C2C12 cell and mouse quadricep muscles after PD stimulation. These results suggested that C2C12 cells could be used as an in vitro cell model for studying the mechanisms of action of PD.

The cytotoxicity of PD towards C2C12 cells is a mixed type of pyroptosis, necroptosis, and autophagy. The crosstalk between these processes is immensely responsible for the death of cells. Various cell death pathways always affect each other. The inhibition of autophagy by a combination of a mechanistic target of rapamycin kinase (mTOR) and a lysosomal inhibitor resulted in RIPK1-dependent necroptosis in human renal carcinoma cell lines [66], while GX15-070-induced autophagy was reported to recruit Fas associated via death domain (FADD)/RIPK1/RIPK3 to the autophagosomal membranes in rhabdomyosarcoma cells [67]. It has been reported that autophagy inhibitor 3-MA enhances caspase-1-dependent pyroptosis in *Shigella*-infected macrophages [68]. These results suggest a bidirectional regulation of autophagy on cell death. The role of autophagy in mediating the adjuvant activity of PD needs further study.

Pyroptosis, a lytic form of cell death, is mediated by excessive activation of caspase-1 or caspase-11/4/5, subsequently a cleavage of pro-IL-1β and IL-18, and an enhanced secretion of IL-6, tumor necrosis factor (TNF)-α, IL-1α, and other inflammatory factors [69]. The activation of caspase-1 is directly mediated through the inflammasome, a multiprotein complex, which consists of members of the NOD-like receptor (NLR) family, including NLRP1, NLRP3, NLRC4, AIM2, or pyrin, and the adaptor ASC, by recruiting pro-caspase-1 and activating the effector caspases through proteolytic cleavage. Many synthetic adjuvants activate the inflammasome, and the NLRP3 is the most common adjuvant target [54]. In vitro results showed that PD also targets the NLRP3 inflammasome, which is similar to the mechanism of action of most adjuvants. It was reported that NLRP3 expression was induced in myeloid cells via NF-κB signaling [70]. In this study, however, PD promoted phosphorylation of p38 and JNK in the MAPK family in the C2C12 cells, while NF-κB phosphorylation was not affected, suggesting cellular differences in inflammasome activation. Collectively, these results demonstrated the involvement of the Ca^2+^−JNK/p38 MAPK−NLRP3 inflammasome−caspase-1 pathway in the pyroptosis and inflammatory response induced by PD in the C2C12 cells. More importantly, both NLRP3 inhibitor MCC950 and caspase-1 inhibitor Ac-YVAD-CMK inhibited PD-induced cytokine production and immune cell recruitment at the injection site, and Ac-YVAD-CMK impaired the adjuvant activity of PD on both antigen-specific cellular and humoral immune responses to OVA in mice.

In this study, PD was found to trigger a remarkable secretion of IL-1β from mouse quadricep muscles, while the IL-1β in the PD-treated C2C12 cells was almost undetectable (data not shown). Actually, the C2C12 myoblasts maintain the ability of differentiation into myotubes [71]. Myoblasts and myotubes constitutively expressed *Tlr1−9*; however, *Tlr2*, *Tlr3*, and *Tlr4* were significantly increased upon differentiation [72], which implies that skeletal muscle cells may be more sensitive to PD. On the other hand, the presence of various types of cells, such as the fibroblasts, innate immune cells in situ, and infiltrating cells at the injection site contributed to the secretion of IL-1β directly or indirectly. These might explain the difference in IL-1β levels induced by PD between the C2C12 myoblasts and mouse quadricep muscles.

PD was showed to induce the pyroptosis and inflammatory response in the C2C12 cells through the Ca^2+^−JNK/p38 MAPK−NLRP3 inflammasome−caspase-1 pathway. However, how PD affects intracellular Ca^2+^ levels, that is, the upstream mechanism of Ca^2+^, has not been elucidated. Recent studies have shown that QS-21 was internalized via a cholesterol-dependent mechanism, and eventually transferred to and concentrated densely in lysosomes where it destroyed lysosomal homeostasis, leading to lysis of membranes and leakage of lysosomal contents [15]. The mitochondria are destabilized by the stimulus, and then releasees the contents such as Ca^2^. Both lysosomes and mitochondria are involved in the regulation of Ca^2+^ homeostasis [73,74]. Therefore, PD could also elevate Ca^2+^ levels through a similar mechanism. In addition to Ca^2+^, ROS and cAMP have also been reported to promote phosphorylation of JNK and p38 MAPK, and directly activate the inflammasome [75,76]. PD was also found to significantly elevate the levels of intracellular ROS and cAMP in the C2C12 cells (Figure S6). The role of ROS and cAMP in mediating adjuvant activity of PD is an issue that warrants further evaluation.

The proinflammatory chemokines and cytokines play a key role in the recruitment of immune cells at the injection site to induce an adaptive immune response. CCL3 promotes the recruitment of monocytes and immature DCs [77], while CXCL2 recruits the neutrophils [78]. IL-6 plays a vital role in muscle immunity and regeneration. IL-6 regulates the transition from neutrophil recruitment to monocyte recruitment in the inflammatory response, and directly or indirectly regulates the local inflammatory response in vivo [79,80]. IL-6 also has a unique function in mediating damage repair in muscle tissues [81]. IL-1β is involved in the acute inflammatory response and immune regulation, and is used as an indicator for pyroptosis. However, the excessive inflammatory responses lead to toxicity and disease. In this study, IL-6, IL-1β, CCL3, and CXCL2 induced by PD at the injection site were transient, as the maximal levels presented at 6 h and a downward trend was exhibited at 12 h following injection. Therefore, the local transient toxicity and inflammatory responses induced by PD meet the safety requirements for adjuvant development [82,83]. Meanwhile, it also suggested that PD could exert adjuvant activity through inducing the production of these chemokines and cytokines from the pyroptotic cells.

**Figure 9:**
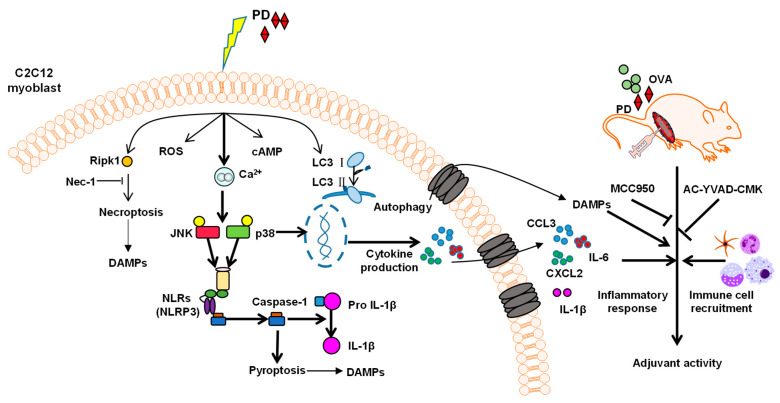
Proposed mechanisms of adjuvant action of PD. PD can induce necroptosis, pyroptosis, and autophagy in C2C12 myoblasts. Caspase-1-dependent pyroptosis mediated the inflammatory response in the C2C12 cells and was regulated by Ca^2+^−JNK/p38 MAPK−NLRP3 inflammasome signaling. The inflammatory cytokines and DAMPs released from the dying cell promoted the recruitment of immune cells into the injection site, leading to overall strongly enhanced adaptive immune responses in vivo.

In conclusion, C2C12 myoblasts were for the first time investigated as an in vitro model in exploring the mechanism of action of an adjuvant. Our experimental data revealed that PD induced pyroptosis and an inflammatory response in C2C12 myoblasts through the Ca^2+^−JNK/p38 MAPK−NLRP3 inflammasome−caspase-1 pathway. Furthermore, it was proposed that PD could exert adjuvant activity through inducing the secretion of inflammatory cytokines and the recruitment of immune cells at the local tissues via the NLRP3 inflammasome−caspase-1 pathway (Figure 9). This study might provide insights into the molecular mechanisms of the adjuvant action of PD.

## Data Availability

Data presented in this study are contained within this article and in the supplementary materials, or are available upon request to the corresponding author.

## Supplementary Materials

The following are available online at https://www.mdpi.com/article/10.3390/cells11010134/s1, Figure S1: The flow diagram of the proportion of different staining states in C2C12 cells using Annexin V-FITC/PI staining, Figure S2: The levels of intracellular free calcium in C2C12 cells treated with PD, Figure S3: The effects of PD on the protein expression levels of caspase-1 and IL-1β in C2C12 cells. Figure S4: The flow cytometry gating strategy of recruited immune cells into the quadricep muscles, Figure S5: Effects of PD on the mRNA expression levels of Th1/Th2 cytokines in OVA-stimulated splenocytes from the OVA-immunized mice, Figure S6: Effects of PD on the levels of intracellular ROS and cAMP in C2C12 cells, Table S1: Sequences of primers used for RT-qPCR.
