# Supplementary material for: Caspase-1-Dependent Pyroptosis Mediates Adjuvant Activity of Platycodin D as an Adjuvant for Intramuscular Vaccines
Source: Cells. 2022 Jan 1;11(1):134. doi: 10.3390/cells11010134 (PMC8750424; doi:10.3390/cells11010134)
Supplement: Supplementary file 1 [file cells-11-00134-s001.zip › cells-1506748-supplementary.pdf]

Supplementary Materials

Caspase-1–dependent pyroptosis mediates adjuvant activity of platycodin D as an adjuvant for intramuscular vaccines

Liyan Zhu, Ziyi Han, Yanfei He , and Hongxiang Sun *

College of Animal Sciences, Zhejiang University, Hangzhou, Zhejiang 310058, China; juliet@zju.edu.cn (L.Y.Z.); 15222671162@163.com (Z.Y.H.); 11717039@zju.edu.cn (Y.F.H.); sunhx@zju.edu.cn (H.X.S)

* Correspondence: sunhx@zju.edu.cn; Tel.: +86-570-8898-2091

A

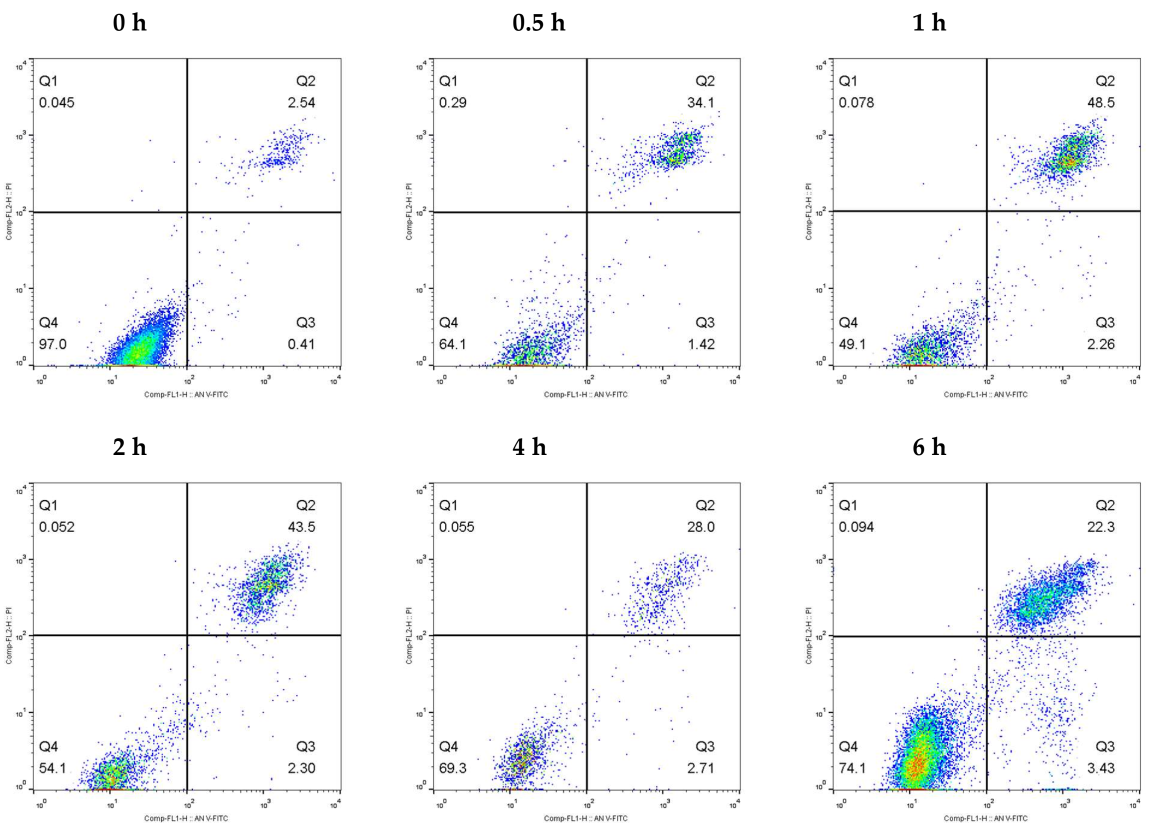
0 h
Q1 0.045
Q2 2.54
Q4 97.0
Q3 0.41
0.5 h
Q1 0.29
Q2 34.1
Q4 64.1
Q3 1.42
1 h
Q1 0.078
Q2 48.5
Q4 49.1
Q3 2.26
2 h
Q1 0.052
Q2 43.5
Q4 54.1
Q3 2.30
4 h
Q1 0.055
Q2 28.0
Q4 69.3
Q3 2.71
6 h
Q1 0.094
Q2 22.3
Q4 74.1
Q3 3.43
Comp-FL1-H :: AN V-FITC
Comp-FL2-H :: PI

B

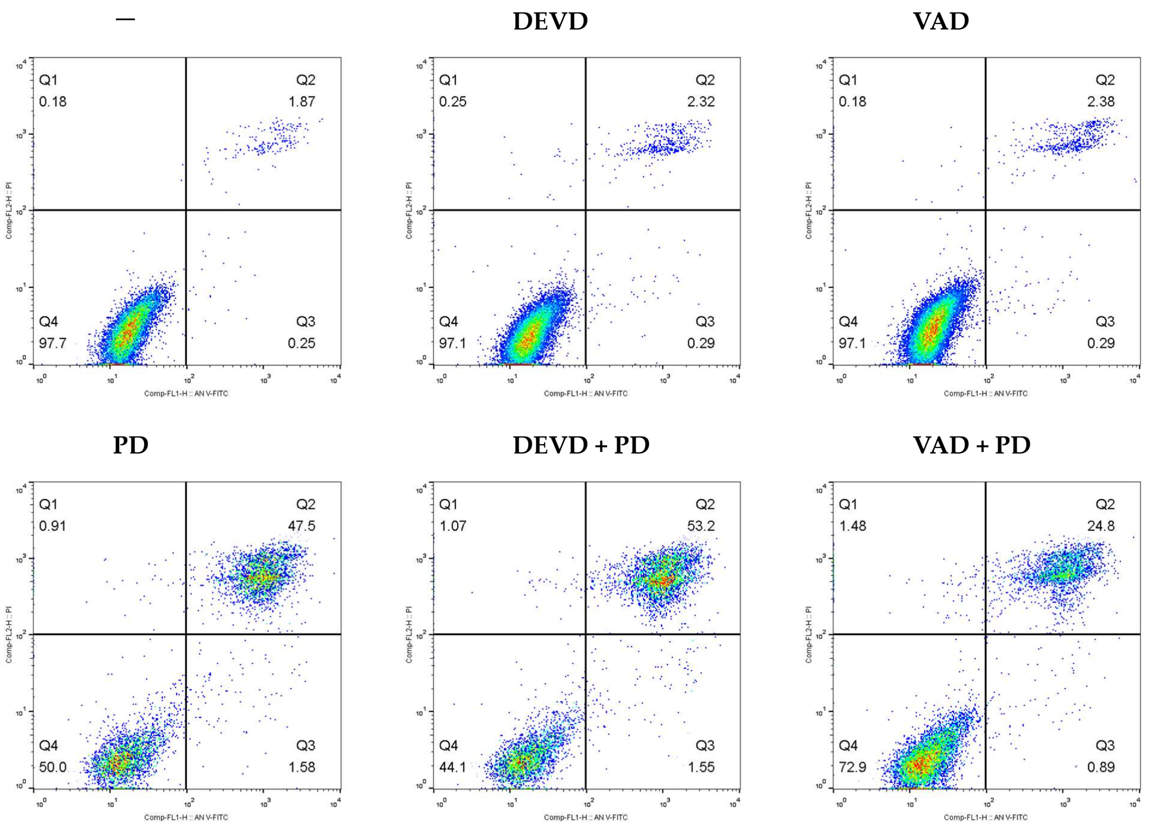
—
Q1 0.18
Q2 1.87
Q4 97.7
Q3 0.25
DEVD
Q1 0.25
Q2 2.32
Q4 97.1
Q3 0.29
VAD
Q1 0.18
Q2 2.38
Q4 97.1
Q3 0.29
PD
Q1 0.91
Q2 47.5
Q4 50.0
Q3 1.58
DEVD + PD
Q1 1.07
Q2 53.2
Q4 44.1
Q3 1.55
VAD + PD
Q1 1.48
Q2 24.8
Q4 72.9
Q3 0.89
Comp-FL1-H :: AN V-FITC
Comp-FL2-H :: PI

C

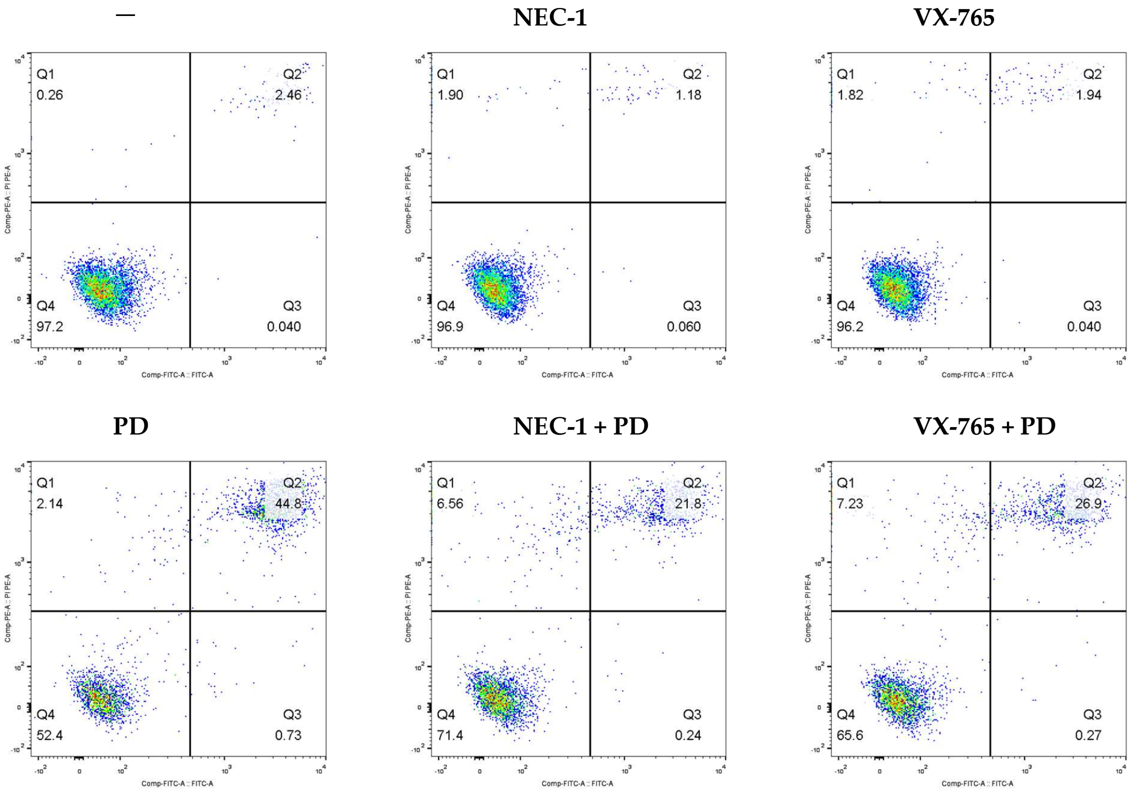

Figure S1. The flow diagram of proportion of different staining states in C2C12 cells using Annexin V-FITC/PI staining. Q1: necrotic cells; Q2: necrotic-like cells; Q3: early apoptotic cells; Q4: intact cells. (**A**) C2C12 cells were stimulated with PD (25 μM) at indicated time. (**B**,C) C2C12 cells were pretreated with indicated inhibitors before PD (25 μM) stimulation for 1 h.

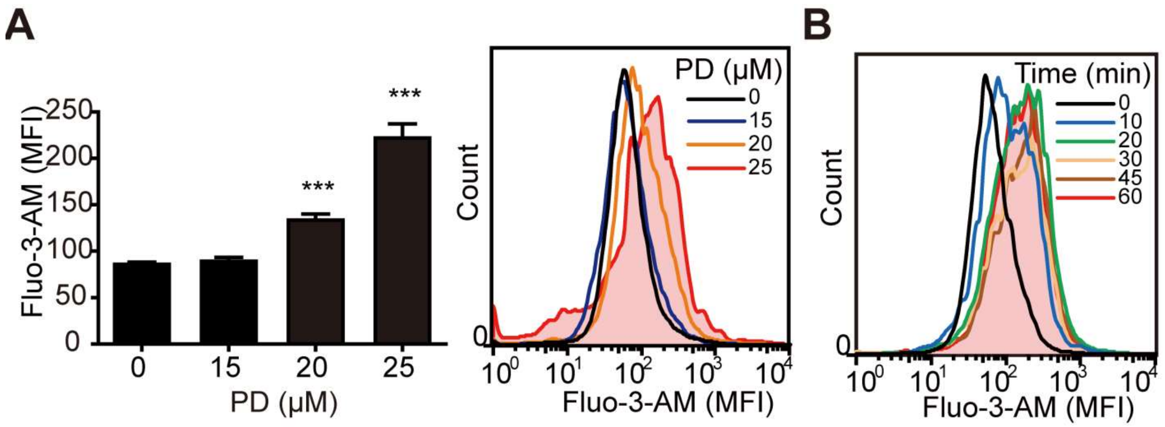

Figure S2. The levels of intracellular free calcium in C2C12 cells treated with PD at 0−25 μM for 20 min (**A**) and at 25 μM for 0−60 min (**B**) by flow cytometry. The histograms shown were representative of three independent experiments.

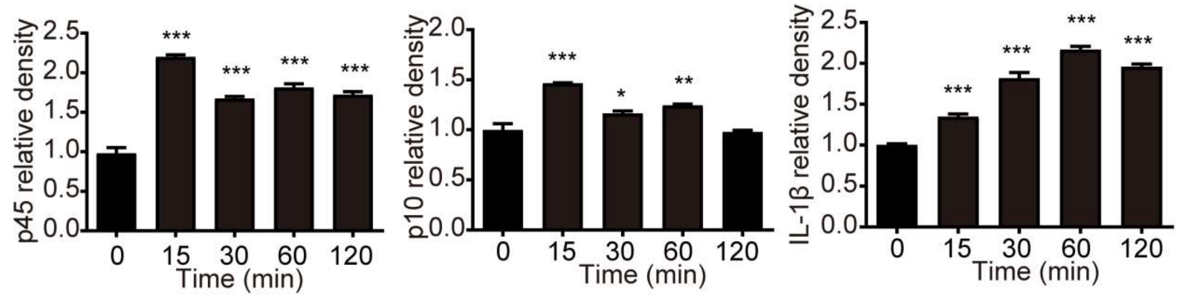

Figure S3. The effects of PD on the protein expression levels of caspase-1 and IL-1β in C2C12 cells. C2C12 cells were stimulated with PD (25 μM) at the indicated time, and the activated caspase-1 and mature IL-1β protein levels were analyzed by Western blotting. p45, pro-caspase-1; p10, activated caspase-1. Quantitative results of mature IL-1β, p45, and p10 in Figure 6C (n = 3, 'n' is the number of replicates). Data were presented as mean ± SD. (*) $p < 0.05$, (**) $p < 0.01$, (***) $p < 0.00$.

A

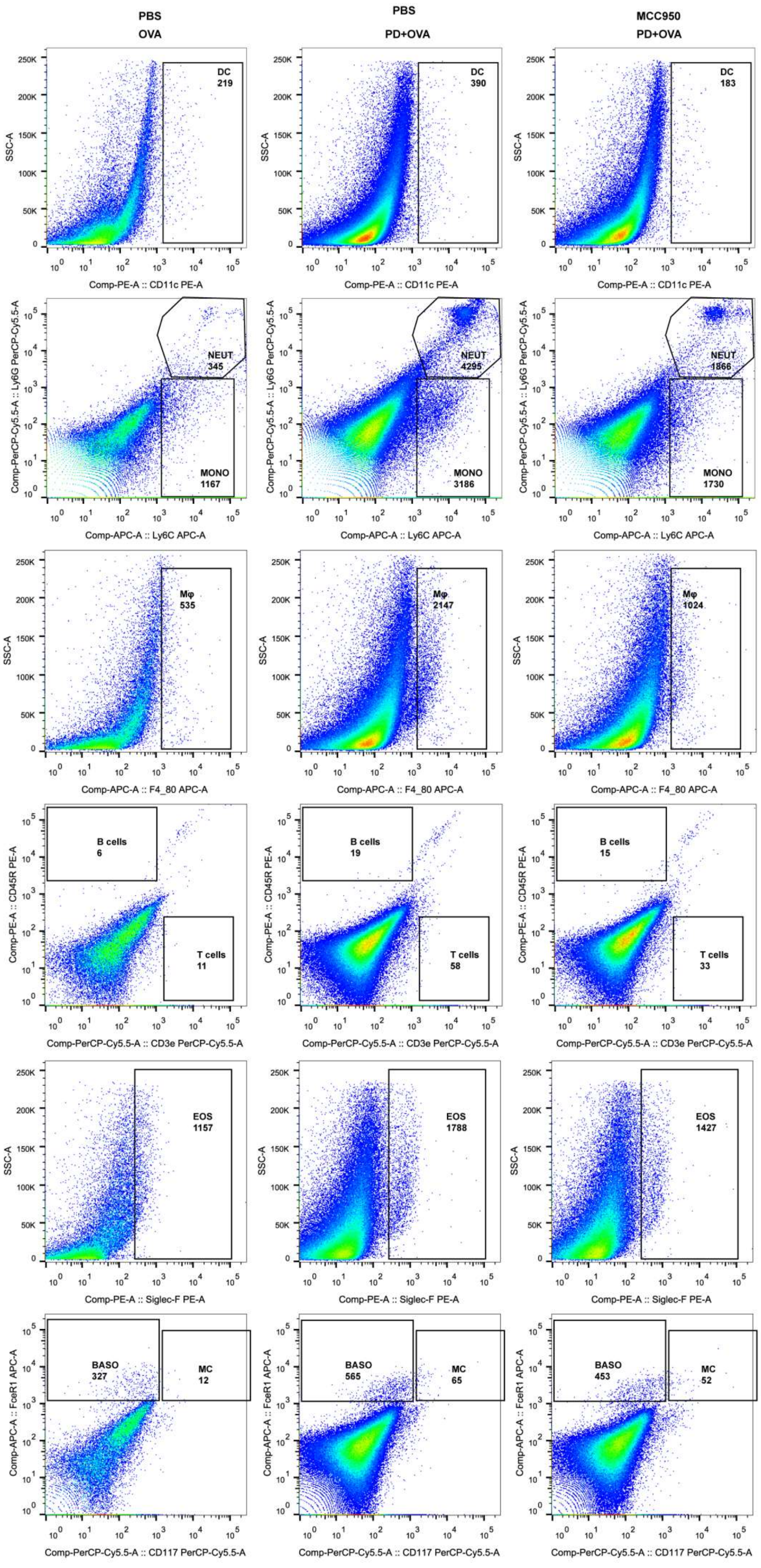
PBS
OVA
PBS
PD+OVA
MCC950
PD+OVA
DC 219
DC 390
DC 183
SSC-A
Comp-PE-A :: CD11c PE-A
NEUT 345
MONO 1167
NEUT 4295
MONO 3186
NEUT 1866
MONO 1730
Comp-PerCP-Cy5.5-A :: Ly6G PerCP-Cy5.5-A
Comp-APC-A :: Ly6C APC-A
Mφ 535
Mφ 2147
Mφ 1024
Comp-APC-A :: F4_80 APC-A
B cells 6
T cells 11
B cells 19
T cells 58
B cells 15
T cells 33
Comp-PE-A :: CD45R PE-A
Comp-PerCP-Cy5.5-A :: CD3e PerCP-Cy5.5-A
EOS 1157
EOS 1788
EOS 1427
Comp-PE-A :: Siglec-F PE-A
BASO 327
MC 12
BASO 565
MC 65
BASO 453
MC 52
Comp-APC-A :: FceR1 APC-A
Comp-PerCP-Cy5.5-A :: CD117 PerCP-Cy5.5-A

B

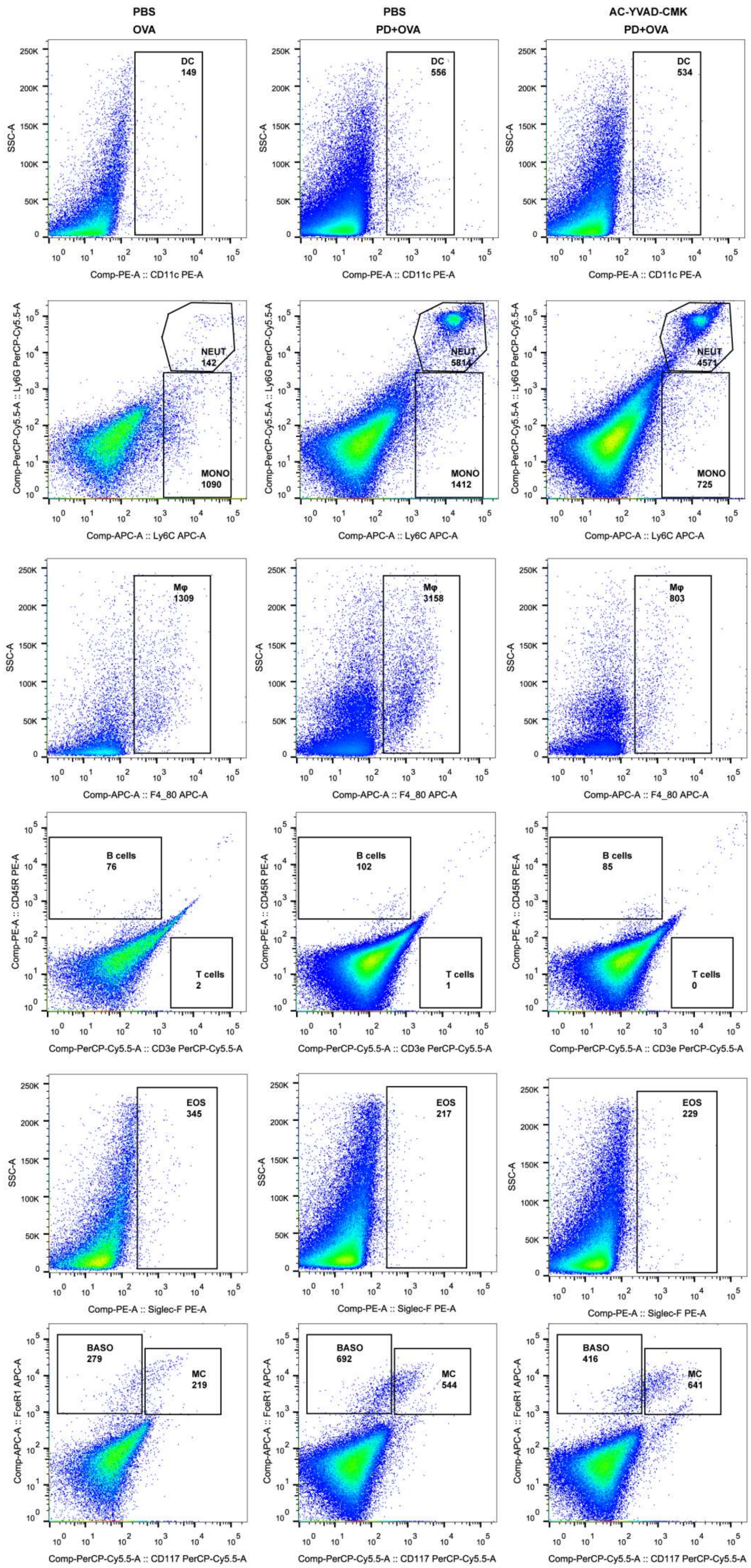
PBS
OVA
PBS
PD+OVA
AC-YVAD-CMK
PD+OVA
DC
149
DC
556
DC
534
SSC-A
Comp-PE-A :: CD11c PE-A
NEUT
142
NEUT
5814
NEUT
4571
MONO
1090
MONO
1412
MONO
725
Comp-PerCP-Cy5.5-A :: Ly6G PerCP-Cy5.5-A
Comp-APC-A :: Ly6C APC-A
Mφ
1309
Mφ
3158
Mφ
803
Comp-APC-A :: F4_80 APC-A
B cells
76
B cells
102
B cells
85
T cells
2
T cells
1
T cells
0
Comp-PE-A :: CD45R PE-A
Comp-PerCP-Cy5.5-A :: CD3e PerCP-Cy5.5-A
EOS
345
EOS
217
EOS
229
Comp-PE-A :: Siglec-F PE-A
BASO
279
MC
219
BASO
692
MC
544
BASO
416
MC
641
Comp-APC-A :: FceR1 APC-A
Comp-PerCP-Cy5.5-A :: CD117 PerCP-Cy5.5-A

Figure S4. The flow cytometry gating strategy of recruited immune cells into the quadriceps muscles. Mice were injected *i.m.* with MCC950 (**A**, 3 mg/kg, 30 min) or AC-YVAD-CMK (**B**, 1 mg/kg, 1 h) and with PD (50 μg) in quadriceps muscles at the indicated time interval. After 24 h, the quadriceps muscle tissues were collected and assayed for the recruitment of various immune cells. DC, dendritic cell; MONO, monocyte; NEUT, neutrophil; Mφ, macrophage; EOS, eosinophil; BASO, basophil; MC, mast cell.

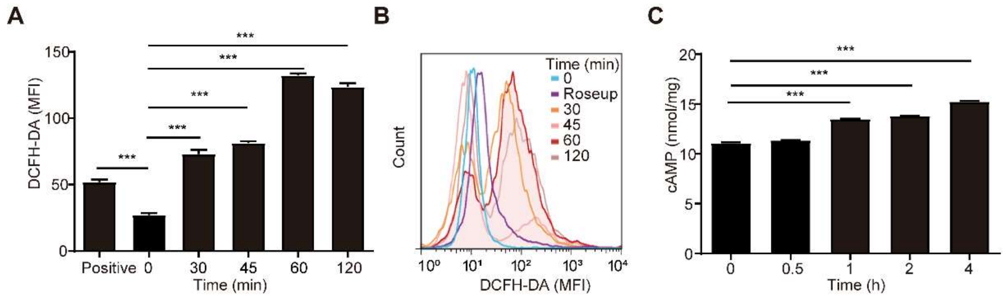

Figure S5. Effects of PD on the levels of intracellular ROS and cAMP in C2C12 cells. (**A**, **B**) The cells were incubated with PD at 25 μM for 0–120 min, and then collected to detect the levels of ROS using 2′,7′-dichlorofluorescein diacetate (DCFH-DA) by flow cytometry. The histograms were representative of three independent experiments. (**C**) The cells were incubated with PD at 25 μM for 0–4 h, and then collected to detect the levels of cAMP using ELISA kit. Data were presented as mean ± SD (n = 3). (***) $p < 0.001$.

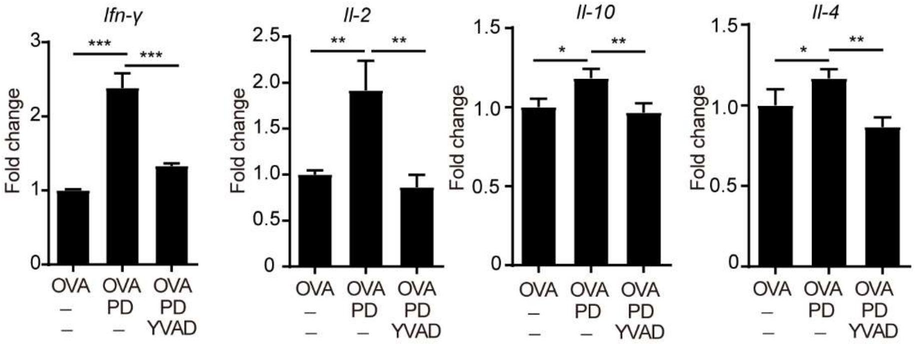

Figure S6. Effects of PD on the mRNA expression levels of Th1/Th2 cytokines in OVA-stimulated splenocytes from the OVA-immunized mice. Mice were injected *i.m.* with Ac-YVAD-CMK at the dose of 1 μg/g for 1 h before immunization. Splenocytes were prepared 2 weeks after the secondary immunization, and then stimulated with OVA (20 μg/ml) for 18 h at 37 °C and 5% CO_2. The collected cells were measured for the mRNA expression levels of *Il-2*, *Il-4*, *Il-10*, and *Ifn-γ* by RT-qPCR. Data were presented as mean ± SD (n = 3). (*) $p < 0.05$, (**) $p < 0.01$, (***) $p < 0.001$, ns not significant.

Table S1. Sequences of primers used for RT-qPCR

Gene	Forward (5′- 3′)	Reverse (5′- 3′)	Product (bp)
Gapdh	AGCCTCGTCCCGTAGACAA	AATCTCCACTTTGCCACTGC	104
Il-6	ACAACCACGGCCTTCCCTACTT	CACGATTTCCCAGAGAACATGTG	129
Il-1β	TTGACAGTGATGAGAATGACCTG	GCTCTTGTTGATGTGCTGCT	137
Ccl-3	ATTCCTGCCACCTGCATAGCT	AGTCCCTCGATGTGGCTACTTG	68
Cxcl2	CTGAACAAAGGCAAGGCTAA	GCACATCAGGTACGATCCAG	125
Caspase-1	CGTACACGTCTTGCCCTCAT	AACTTGAGCTCCAACCCTCG	72
Il-18	TCAGCTGGGAAAACTCAGGA	TGGGAACAGCCAGTGTTCAG	119
Ptgs2	GCAGATGACTGCCCAACTC	CAGGGATGAACTCTCTCCGT	103
Nlrc4	AGAATCGCTATGCTCTGGGC	AAGGTCGGCTTGCTGATGAA	188
Nlrp3	TGGACCAGGTTCAGTGTGTT	CGGTTGGTGCTTAGACTTGA	118
Aim-2	AAATGCTGTTGTTGACCGGC	GAGTGTGCTCCTGGCAATCT	101
Caspase-3	GTCATCTCGCTCTGGTACGG	CACACACACAAAGCTGCTCC	169
Caspase-8	GCAAGACTGGAGCATGAATCTG	CACCCCATTCTGCTGACTCAC	143
Caspase-9	TTGGGCGCAAAGGTTAAAGC	GGAGCCTTCAACAATCACAGC	139
Bax	CTCAAGGCCCTGTGCACTAA	CACGGAGGAAGTCCAGTGTC	73
Bcl-2	CCACCTGTGGTCCATCTGAC	CAATCCTCCCCCAGTTCACC	175
Mlkl	CCGGACAGCAAAGAGCACTA	TCCAGTGGCAATTTCCCAGA	145
Ripk1	GGTCAAATTCAGAACAACCTGGA	CACACTGCGATCATTCTCGT	122
Ripk3	ACACGGCACTCCTTGGTATC	CCGAACTGTGCTTGGTCATA	168
Il-2	CCCAAGCAGGCCACAGAATTGAAA	AGTCAAATCCAGAACATGCCGCAG	81
Ifn-γ	TCTTGAAAGACAATCAGGCCATCA	GAATCAGCAGCGACTCCTTTTCC	233
Il-4	CAAACGTCCTCACAGCAACG	CTTGGACTCATTCATGGTGC	203
Il-10	GCTCTTACTGACTGGCATGAG	CGCAGCTCTAGGAGCATGTG	105
